# Impact of the COVID-19 pandemic on oncological care in Germany: rapid review

**DOI:** 10.1007/s00432-023-05063-9

**Published:** 2023-07-29

**Authors:** Karina Karolina De Santis, Stefanie Helmer, Benjamin Barnes, Klaus Kraywinkel, Maren Imhoff, Roxana Müller-Eberstein, Mathia Kirstein, Anna Quatmann, Julia Simke, Lisa Stiens, Lara Christianson, Hajo Zeeb

**Affiliations:** 1 Department of Prevention and Evaluation, Leibniz Institute for Prevention Research and Epidemiology- BIPS, Bremen, Germany; 2 Faculty 11 Human and Health Sciences, University of Bremen, Bremen, Germany; 3 German Center for Cancer Registry Data, Robert Koch Institute (RKI), Berlin, Germany

**Keywords:** Cancer, Neoplasms, Oncological care, Oncology service, COVID-19, Germany

## Abstract

**Objectives:**

The COVID-19 pandemic affected medical care for chronic diseases. This study aimed to systematically assess the pandemic impact on oncological care in Germany using a rapid review.

**Methods:**

MEDLINE, Embase, study and preprint registries and study bibliographies were searched for studies published between 2020 and 2 November 2022. Inclusion was based on the PCC framework: population (cancer), concept (oncological care) and context (COVID-19 pandemic in Germany). Studies were selected after title/abstract and full-text screening by two authors. Extracted data were synthesized using descriptive statistics or narratively. Risk of bias was assessed and summarized using descriptive statistics.

**Results:**

Overall, 77 records (59 peer-reviewed studies and 18 reports) with administrative, cancer registry and survey data were included. Disruptions in oncological care were reported and varied according to pandemic-related factors (e.g., pandemic stage) and other (non-pandemic) factors (e.g., care details). During higher restriction periods fewer consultations and non-urgent surgeries, and delayed diagnosis and screening were consistently reported. Heterogeneous results were reported for treatment types other than surgery (e.g., psychosocial care) and aftercare, while ongoing care remained mostly unchanged. The risk of bias was on average moderate.

**Conclusions:**

Disruptions in oncological care were reported during the COVID-19 pandemic in Germany. Such disruptions probably depended on factors that were insufficiently controlled for in statistical analyses and evidence quality was on average only moderate. Research focus on patient outcomes (e.g., longer term consequences of disruptions) and pandemic management by healthcare systems is potentially relevant for future pandemics or health emergencies.

**Supplementary Information:**

The online version contains supplementary material available at 10.1007/s00432-023-05063-9.

## Introduction

The COVID-19 pandemic contributed to rapid reorganization of medical care for chronic diseases, such as cancer in Germany and elsewhere (Scheidt-Nave et al. 2021). Emergency care for COVID-19 cases was prioritized, while other hospital admissions, planned medical treatments or existing care for chronic diseases were adapted to the pandemic conditions, postponed or cancelled. Disruptions in oncological care were reported in Europe, North America and other world regions (The Lancet Oncology 2020). Missed or delayed cancer screening or treatment could lead to an increase in symptomatic cancer patients (Jones et al. 2020) or worse outcomes (Raphael et al. 2016) as well as more cancer-related deaths (Joung et al. 2022).

Conflicting results regarding oncological care were reported since the onset of the COVID-19 pandemic in Germany. Perceived disruptions in medical care for chronically ill or people of advanced age (Heidemann et al. 2020) and disruptions in oncological care were reported (Weisel et al. 2020). Specifically, a scoping review covering the first few months of the COVID-19 pandemic reported a reduced number of consultations, inpatient admissions and tumor diagnostic procedures as well as fewer primary surgeries for some cancer entities in Germany (Scheidt-Nave et al. 2021). However, these disruptions were not perceived as critical and probably affected only a small number of time-critical therapies at the time (Scheidt-Nave et al. 2021). Nearly three years into the pandemic (i.e. in October 2022) a coherent picture of the characteristics and extent of disruptions in oncological care provision is still missing in Germany, partly due to the heterogeneity in data sources and reported data, but also because a comprehensive assessment of disruptions is possible only after sufficient time has passed. Thus, this study aimed to systematically assess the impact of the COVID-19 pandemic on oncological care in Germany using rapid review methods.

## Materials and methods

### *Review designand protocol*

This study is a rapid review based on the methodological recommendations from Cochrane (Garritty et al. 2021). Rapid review methodology is appropriate to address urgent health issues with high priority (Garritty et al. 2021). Since our aim was to map the existing literature by broadly focusing on any aspect of oncological care (e.g., data sources, sample and cancer types, periods of data collection and clinical settings), we follow the recommendations for scoping reviews to chart and synthesize the data (Pollock et al. 2022; Tricco et al. 2018). The reporting adheres to the Preferred Reporting Items for Systematic Reviews and Meta-Analyses Extension for Scoping Reviews (PRISMA-ScR) checklist (Tricco et al. 2018); Supplementary Information, Table S1. A protocol for this review was prospectively registered at the Open Science Framework (osf.io/f2dnj). There were no changes between the protocol and this review.

### *Eligibilitycriteria*

Inclusion was based on the PCC framework: (1) population: cancer patients (any age), people eligible for screening or healthcare professionals in oncology, (2) concept: oncological care (delivery and utilization), (3) context: the COVID-19 pandemic in Germany (i.e., March 2020 through October 2022 with or without pre-pandemic comparison period), (4) study type: primary studies published in peer-reviewed journals with any study design and data type, reports without peer-review (e.g., health insurance claims data) or preprints; in English or German language and available in full-text. Conference papers, dissertations, reviews, and commentaries were excluded.

### Information sources

The information sources were bibliographic databases (MEDLINE and Embase), bibliographies of included studies, online registers of COVID-19 studies or preprints, and reports without peer-review (via relevant organization websites). Syntax and search strategies are reported in Supplementary Information, Tables S2-S4.

### Search strategy

The search syntax was developed and calibrated within the team. MEDLINE and Embase were searched on 2 November 2022 by experienced librarians while the other information sources were searched through 15 November 2022 by the authors. Search results were stored and managed in EndNote X9 and Covidence.

### Selection of sources of evidence

Title/abstract and full-text screening were performed independently by two authors and relevant studies were selected by consensus.

### Data charting

Peer-reviewed studies were coded by one author and checked by another author using a self-developed data charting form in Excel 10. The data were coded into pre-defined categories or charted into themes that inductively emerged from studies according to recommendations for scoping reviews (Pollock et al. 2022). Reports without peer-review were narratively coded by one author.

### Data items

Data items included bibliographic information, study characteristics (study design, data source, region in Germany, data collection period), sample characteristics (sample type, cancer type), oncological care (care aspects, study outcomes, factors associated with care) and evidence gaps. Based on descriptive patterns in study outcomes, oncological care was classified as either ‘no changes in care’ or ‘disruptions in care’.

### Critical appraisal (risk of bias assessment)

Based on recommendations for rapid reviews (Garritty et al. 2021), we performed a risk of bias assessment to evaluate the evidence quality in peer-reviewed studies using validated instruments from Cochrane and JBI or additional bespoke instruments for specific study types (e.g., modelling studies; Supplementary Information, Table S5). Each study was independently assessed by two authors and any disagreements were resolved by consensus. In general, items indicating a low risk of bias (i.e., fulfilled or not applicable items) were scored as 1 and items indicating a high risk of bias (i.e., not fulfilled, not reported or unclearly reported items) were scored as 0. For example, an item was fulfilled and scored as 1 if a study controlled for confounders in a statistical analysis. A mean risk of bias score was computed for all studies as the sum of all items rated 1 out of all items divided by the number of studies.

### Data synthesis

We synthesized the data using descriptive statistics or narrative descriptions of common themes.

### Stakeholder involvement

We discussed the data items with a relevant stakeholder (a counselor at the Bremen Cancer Society) to ensure that we address the most important aspects of oncological care in this rapid review.

## Results

### Study selection

**Fig. 1:**
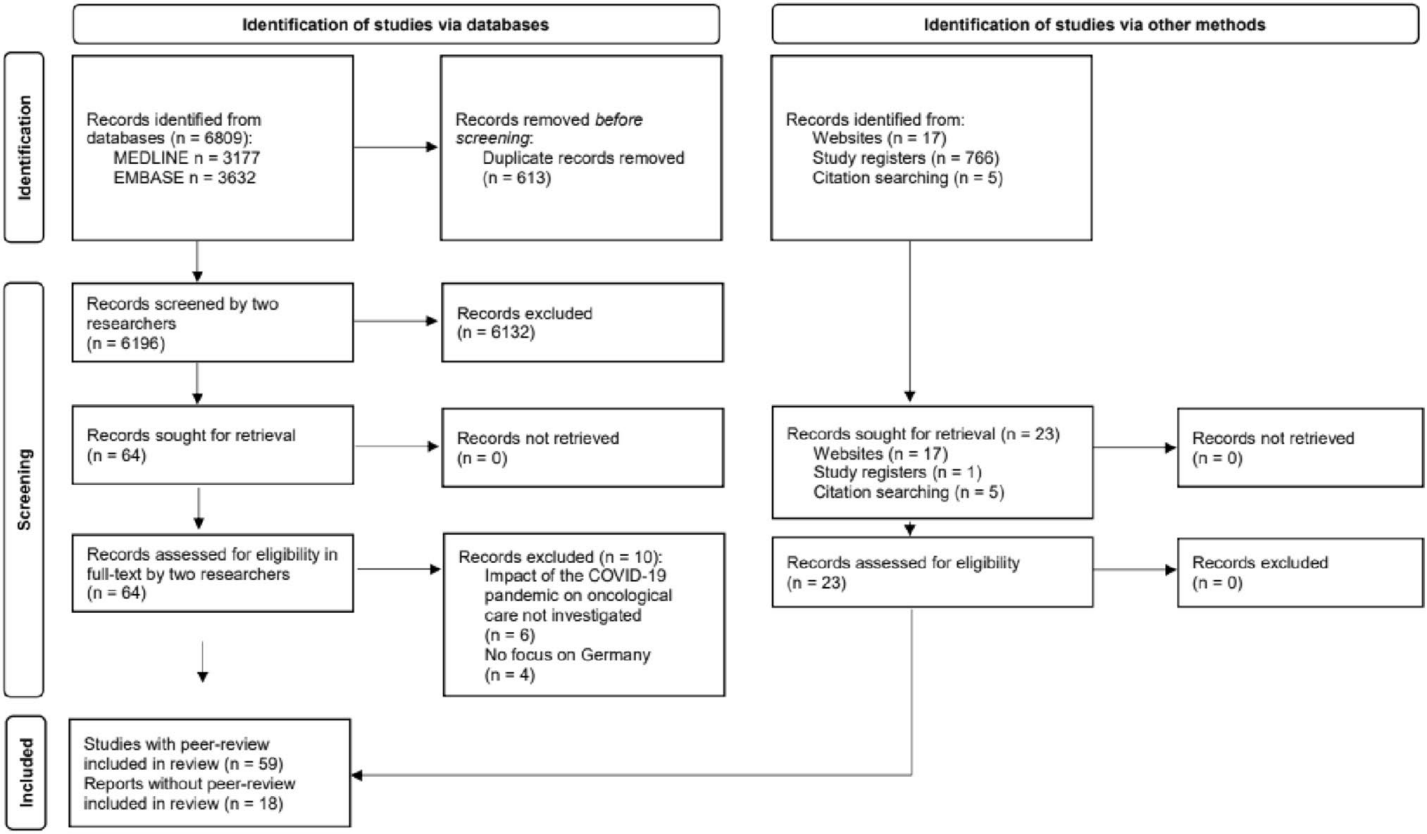
Study selection (PRISMA flow diagram)

From 6196 records identified via database searches and 788 records identified via other methods, 77 records met the inclusion criteria (Fig. 1). These included 59 peer-reviewed studies (Arndt et al. 2022; Balakirski et al. 2022; Balk et al. 2022; Bartella et al. 2021; Beller et al. 2022; Bollmann et al. 2021; Brunner et al. 2020; Buntzel et al. 2020, 2021; Dienemann et al. 2021; Diers et al. 2021, 2022; Donath et al. 2021; Eckford et al. 2021; Erdmann et al. 2021, 2022; Fauser et al. 2022; Gremke et al. 2022; Griewing et al. 2022a, b; Gschnell et al. 2021; Haier et al. 2022a, b, c; Hajek et al. 2021; Harke et al. 2020, 2022; Heimes et al. 2021; Holzel et al. 2022; Hunger et al. 2022; Jacob et al. 2021, 2022; Jördens et al. 2021; Justenhoven and Rieger 2022; Kaltofen et al. 2022; Kapsner et al. 2020; Kirchberg et al. 2021; Kleemann et al. 2022; Kourtidis et al. 2022; Kuhlen et al. 2020; Matuschek et al. 2020; Medenwald et al. 2022; Micek et al. 2022; Michalowsky et al. 2021; Piontek et al. 2021; Reichardt et al. 2021; Riemann et al. 2021; Rupa et al. 2020; Schuz et al. 2022; Stang et al. 2020; Stos et al. 2020; Struck et al. 2022; Teuscher et al. 2022; Voigtlander et al. 2021; Vu et al. 2022; Walter et al. 2021, 2022; Wang et al. 2020; Ziegler et al. 2022) and 18 reports without peer-review (Acar et al. 2021; Deutsche Krebsgesellschaft Deutsche Krebshilfe Deutsches Krebsforschungszentrum 2021a, b; Fröhling and Arndt 2020; Günster et al. 2020; Heidt et al. 2021; Hermes-Moll et al. 2021; Klinische Krebsregister Sachsen 2022; Klinisches Krebsregister für Brandenburg und Berlin 2022a, b; Mangiapane et al. 2021, 2022; Mostert et al. 2021; Rückher and Pflüger 2022; Tillmanns et al. 2022; Wissenschaftliches Institut der AOK WIdO 2021, 2022; Zok 2021). A list of excluded studies is reported in Supplementary Information, Table S6. All data are reported in Supplementary Information, Tables S7-S8.

### Bibliographic characteristics

The 59 peer-reviewed studies were published between 2020 and 2022. The studies reported either no conflicts of interest due to funding (43/59) or did not report the sources of funding (16/59).

### Study characteristics

**Fig. 2:**
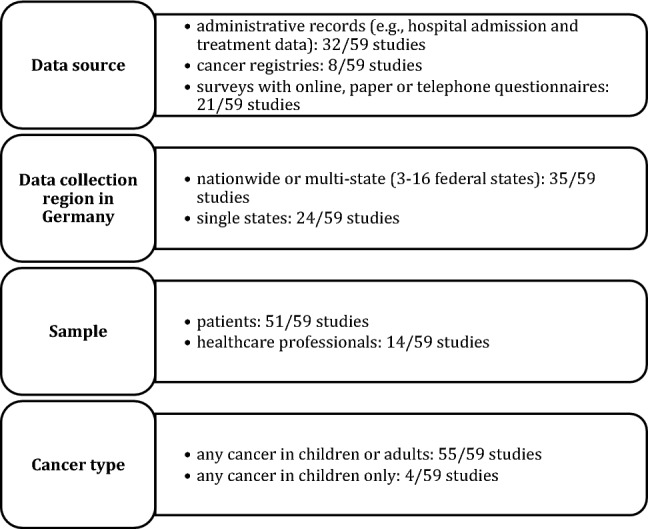
Study characteristics

All 59 peer-reviewed studies were based on administrative, cancer registry or survey data in Germany (Fig. 2). Most studies included patients of any age and with any cancer type (already diagnosed or in the screening or detection stage).

### Oncological care during the COVID-19 pandemic

Oncological care was assessed either solely over the course of the COVID-19 pandemic (i.e., March 2020 onwards) in all studies or in relation to different pre-pandemic periods (i.e., before March 2020) in 39/59 studies. Oncological care was delivered in any clinical setting based on location (e.g., general hospital, specialized clinic, medical practice, rehabilitation facility) and provision (i.e., in- or outpatient care). Five care aspects inductively emerged from the studies:

1. Any (general or unspecified) care: consultations, appointments, hospitalizations, and related details (e.g., length of hospital stay, restrictions in care, mental burden)
2. Diagnosis: screening, incidence or detection of tumor or metastases
3. Treatment: surgery, radiotherapy, systemic therapy or psychosocial care
4. Aftercare: follow-up treatment or rehabilitation
5. Other (specific) care: palliative care and related outcomes (survival rate or mortality)

Since this review is based on data from one country and one disease group (i.e., cancer) there is a risk that the same patients or healthcare professionals were included in multiple studies. To reduce double counting, we clustered the studies that used the same or similar data sources (e.g., same hospital groups or databases) or the same regions in Germany into groups. We then assessed and reported oncological care within each group of studies.

Based on 32 studies with administrative data (cited in Supplementary Information, Table S9) and eight studies with cancer registry data (cited in Supplementary Information, Table S10) collected nationwide or regionally, oncological care was temporarily disrupted throughout 2020–2021, although some studies did not report any changes relative to 2019 or earlier (Table 1). Disruptions were reported during periods of high restrictions and some recovered after restrictions were relaxed. Most disruptions were detected for low-risk patients (e.g., delays in screening and non-urgent surgery) while ongoing treatment was unchanged. Most consistent disruptions were reported for any general or unspecified care (e.g., patient volume), diagnosis (e.g., detection or screening) and treatment (e.g., surgery). Treatment types other than surgery (e.g., psychosocial care) and aftercare (e.g., follow-up treatment) were less often assessed and there were mixed results. Any disruptions in oncological care depended on pandemic stage (i.e., periods of high or low restrictions), institution type (e.g., hospitals or outpatient facilities), region in Germany, cancer type and stage, and patient characteristics. Furthermore, 21 studies with survey data (cited in Supplementary Information, Table S11) confirmed the trends seen in studies with administrative and registry data (Table 1). Patients and healthcare professionals reported fewer consultations and disruptions in appointments (e.g., delays in screening), reduced access to clinical facilities for patients and accompanying persons and higher mental burden related to treatment uncertainties. Healthcare professionals reported higher workload, mental burden and disruptions in clinical management (e.g., changes in clinical processes, limited resources and communication with patients).

**Table 1:** Oncological care during the COVID-19 pandemic in Germany

	Administrative data	Cancer registry data	Survey data
Studies	32/59	8/59	21/59
Data sources	Hospitals (admission and treatment data) Clinical practices Disease databases Insurance providers	6 Cancer registries	16 Surveys (with online, paper or telephone questionnaires) of patients or healthcare professionals 54–1231 participants/survey
Time period	2020–2021 vs. < 2020	2020–2021 vs. < 2020	2020–2022
Care aspects	Most consistent trends: ↓ Any care (consultations, admissions) ↓ Diagnosis (detection, screening demand, diagnoses with available screening programs) ↓ Treatment (surgery) Heterogeneous results: ↓ or ↔ Treatment other than surgery ↓ or ↔ Aftercare	↓ or ↔ Diagnosis (detection, incidence, but ↑ incidence childhood cancers) ↓ or ↔ Treatment (surgery, radiotherapy, systematic therapy) ↓ or ↔ Aftercare ↓ Other (predicted survival rates)	Perceptions of patients: ↓ Any care (consultations, access to clinical facilities, mental burden) ↓ Diagnosis (screening) ↓ Treatment (low-risk surgery, psychosocial care) ↔ Treatment (ongoing, advanced, high-risk) ↓ Aftercare Perceptions of healthcare professionals: ↑ Workload ↑ Disruptions in clinical management ↑ Mental burden
Risk of bias	Moderate: mean = 0.58, SD = 0.14, range: 0.25–0.83	Moderate: mean = 0.50, SD = 0.13, range: 0.42–0.75 High (modeling studies): mean = 0.19, SD = 0.09, range: 0.06–0.25	Moderate: mean = 0.48, SD = 0.30, range: 0–1

The arrows indicate disruptions (↓), no changes ( ↔) or increase (↑). The risk of bias was rated on a scale from 0 (highest risk) to 1 (lowest risk).

In addition to peer-reviewed studies, we also included 18 reports without peer-review (cited in Supplementary Information, Table S12) based on claims, survey, cancer registry data, or unsolicited feedback. These resources were published as reports, magazine articles, or press releases. Reports with hospital data mentioned disruptions in admissions, therapeutic colonoscopies and tumor surgeries with every wave of SARS-CoV-2 infections. Such disruptions varied by pandemic phase, treatment setting (inpatient or outpatient) and procedure. The inpatient setting was more heavily affected than the outpatient setting. Reports discussed a shift in resource allocation within hospitals and between patient groups. In contrast, reports with claims data from the outpatient practice sector showed that case numbers remained stable or tended to increase. Reports with cancer registry data were mostly preliminary. The number of cancer screening examinations varied by cancer type and was affected by temporary suspensions and adaptations of screening programs. Reports with survey data indicate that screening-eligible individuals experienced cancellation or postponement of screening appointments by their healthcare providers. Healthcare providers stated that while the pandemic negatively affected some care aspects, such as follow-up and psychosocial care, acute cancer care was maintained at pre-pandemic levels.

### Risk of bias

The risk of bias was on average moderate (Table 1; Supplementary Information, Tables S13-S16). The most important source of the high risk of bias was that confounding factors were inadequately controlled for when assessing or interpreting the changes in oncological care during the COVID-19 pandemic.

### Factors potentially associated with disruptions in oncological care

Studies included in this review suggest that any disruptions in oncological care in Germany during the COVID-19 pandemic were associated with the pandemic itself (i.e., pandemic-related factors) and other (non-pandemic) factors (Table 2). Some of these factors were controlled for in statistical analyses, while others were mentioned by study authors in discussion, limitations or conclusions.

**Table 2:** Factors potentially associated with disruptions in oncological care

Type	Factor	Example of how the factor could be potentially associated with disruptions in oncological care
Pandemic-related factors	Pandemic stage (restrictions)	Reduced patient volume due to restrictions on public life (e.g., physical distancing, suspension of hospital visits) in Germany
	Pandemic development (COVID-19 case numbers)	Reduced patient volume related to pandemic development (i.e., during pandemic waves with high COVID-19 case numbers)
	Pandemic-related reorganization of care	Reduced patient volume due to temporary reorganization of care during pandemic waves (e.g., relocation of care away from hospitals to outpatient clinics, changes in resource allocation, prioritization of COVID-19 patients, staff shortages due to quarantine)
Other (non-pandemic) factors	Patient characteristics	Care provision and utilization depending on patient sociodemographic and clinical characteristics (e.g., delayed care provision due to an overall clinical status that does not require emergency or urgent treatment)
	Cancer details	Care provision and utilization depending on cancer type, stage, symptoms, tumor size and location (e.g., possibility to postpone non-urgent surgery depending on cancer stage)
	Care setting	Care provision and utilization depending on setting location (e.g. reduced patient volume at smaller clinical facilities and in smaller cities) and provision (e.g., reduced patient volume due to care relocation from in- to outpatient care or centralization from multiple facilities to single hospitals)
	Care details	Care provision and utilization depending on care aspect (e.g., surgery or other treatment, aftercare, or psychosocial care)

### Evidence gaps and topics for future research

Based on the included studies, we inductively identified evidence gaps and topics for future research that focus on patient health outcomes and pandemic management by healthcare systems (Table 3).

**Table 3:** Evidence gaps and topics for future research

Type	Evidence gap	Example of a topic for future research
Patient health outcomes	Long-term effects of disruptions in care	Effects of delayed screening or surgery on health outcomes
	Patient education	Importance of screening and consultations for health outcomes
	Wellbeing of patients	Importance of psychosocial care and aftercare for health outcomes
Pandemic management	Adaptation of organizational processes	Measures required to improve the organizational efficiency and patient management during pandemic conditions
	Evidence-based prioritization in medicine	Justification for delaying oncological care due to emergency in another clinical field
	Wellbeing of healthcare professionals	Measures required to reduce workload and mental burden during pandemic conditions

## Discussion

### Overall summary

Consistent with global disruptions in oncological care (The Lancet Oncology 2020), such disruptions in oncological care were also reported in Germany according to 77 records (59 peer-reviewed studies and 18 reports) included in this rapid review. The disruptions varied according to pandemic-related factors (e.g., pandemic stage) and other (non-pandemic) factors (e.g., care details). During higher restriction periods fewer consultations and non-urgent surgeries, and delayed diagnosis and screening were consistently reported. Heterogeneous results were reported for treatment types other than surgery (e.g., psychosocial care) and aftercare, while ongoing care remained mostly unchanged. The risk of bias was on average moderate.

### Extent of disruptions in oncological care during the COVID-19 pandemic

As suggested by others (Dienemann et al. 2021) and based on studies in this review, disruptions in oncological care reported during the COVID-19 pandemic in Germany probably depended on various pandemic-related and other factors, such as patient and disease characteristics, as well as setting and care details. For example, reduced patient volume especially during the high restriction periods might have been due to pandemic-related reduction in utilization of care (Scheidt-Nave et al. 2021) or to pandemic-unrelated reorganization of care within healthcare institutions (Dinkel et al. 2021; Weisel et al. 2020). In Germany, patient volume at specialist clinics could be affected by difficulties in access to care upstream from such clinics. This is because in Germany patients typically obtain a referral for a specialist consultation from their general practitioners. Thus, reduced access to general practitioners during the pandemic might have contributed to fewer referrals to specialist care and thus lower patient volume. In general, it is difficult to establish to what extent the COVID-19 pandemic affected the oncological care because most studies in this review used data from different pandemic periods and lacked detailed data on other factors.

### Future research

Studies included in this review suggest that patient outcomes related to disruptions in oncological care should be investigated in future research. The ethics of prioritization in medicine and resource allocation that contributed to disruptions in care for chronic diseases have already been questioned (Brunner et al. 2020; Eckford et al. 2021). Delays in cancer detection and treatment are associated with detrimental health effects (Alkatout et al. 2021; Hanna et al. 2020) and are predicted to contribute to higher mortality (Maringe et al. 2020). Furthermore, the patient perspective with respect to psychosocial aspects and care expectations during health emergencies needs to be considered in future research (Dinkel et al. 2021). As shown in this review and other studies (Bauerle et al. 2021; Colomer-Lahiguera et al. 2021; Verma et al. 2022; Ziegler et al. 2022), cancer patients reported a high mental burden of the COVID-19 pandemic due to uncertainties regarding their treatment and restrictions in public life (e.g., visiting restrictions, loneliness and reduction in psychosocial support).

Future studies should also evaluate the effectiveness of pandemic management measures in preparation for any future health emergencies (Weisel et al. 2020). Adaptation of organizational processes was identified as an important measure to maintain the usual oncological care provision even in regions with high incidence of SARS-CoV-2 infections in the early stages of the pandemic in Germany (Akuamoa-Boateng et al. 2020). Various measures to prevent SARS-CoV-2 infections were also necessary to reduce potentially detrimental effects of such infections in people with cancer (Tang and Hu 2020). As sufficient data become available, both health consequences and economic implications of such measures need to be evaluated (Goldsbury et al. 2018; von Dercks et al. 2020). Furthermore, as shown in this review and discussed by others (Beller et al. 2022), pandemic management measures should also focus on healthcare providers with the aim to reduce the workload and the mental burden in oncological health professionals that were reported during the COVID-19 pandemic in Germany.

### Strengths and limitations

The main strength of this review is a large volume of the included literature (59 peer-reviewed studies and 18 reports) based on nationwide data from a single country (Germany) with a highly-developed healthcare system. The results of this review show how this healthcare system managed the oncological care under the strain of a worldwide COVID-19 pandemic. Since the COVID-19 pandemic seems to have ended in most parts of the world, the review deals with a historical health event. Thus, the results of this review are potentially relevant for management of any future pandemics and health emergencies. Furthermore, we identified several factors that could be considered when evaluating longitudinal data on the impact of health emergencies in one clinical field on care patterns for chronic diseases in other fields.

There were several limitations in this review. First, study results were difficult to synthesize due to heterogeneous outcomes and data collection periods during which different pandemic-related restrictions were imposed in Germany (Supplementary Information, Table S17). Second, the evidence quality in this review was on average only moderate based on the risk of bias assessment. Factors that might have affected oncological care patterns over time (e.g., changes in screening programs) were inadequately controlled for in descriptive statistical analyses of longitudinal data collected during the COVID-19 pandemic. While administrative and registry data were affected by delays in data entry and lacked detailed clinical and sociodemographic patient characteristics, survey data may have overestimated the disruptions in care (e.g., pediatricians estimated about 40% fewer consultations, while hospital data showed 30% fewer consultations in one survey (Donath et al. 2021)). Third, as a consequence of the first two limitations, this review qualitatively describes any trends in data because we could not estimate the standardized effect sizes and variance. Computation of effect sizes was not possible because absolute rather than relative data were reported (e.g., absolute patient volume without total patient volume admitted to a clinical facility) and comparison time periods before the pandemic were heterogeneous or not included in the study. Thus, it is unclear if small absolute changes in oncological care are clinically meaningful or to what extent they deviate from the natural fluctuation observed in medical care for chronic diseases. A planned meta-analysis was not performed due to heterogeneous samples, data collection and comparison time periods, and outcomes of oncological care. Fourth, it cannot be ruled out that the same patients were included in multiple studies that used nationwide data, similar data collection periods and overlapping cancer and care types. However, such overlap probably had little consequences on our results because we described the outcomes qualitatively. Fifth, this highly sensitive and politicized field of prioritization in medicine could have been affected by publication bias toward studies reporting disruptions in oncological care. While adaptation of organizational processes contributed to effective oncological care provision during the early stages of the pandemic (Akuamoa-Boateng et al. 2020), other studies not reporting any disruptions may not have been published. Thus, the generalizability of the results of this review beyond the 77 included records is unclear.

## Conclusions

Disruptions in oncological care were reported during the COVID-19 pandemic in Germany according to 77 records. Such disruptions depended on factors that were insufficiently controlled for and evidence quality was on average only moderate. Research focus on patient outcomes (e.g., longer term consequences of disruptions) and pandemic management by healthcare systems is potentially relevant for future pandemics or health emergencies.

## Supplementary Information

Below is the link to the electronic supplementary material.

Supplementary file1 (ZIP 764 KB)

## Data Availability

All data reported in this review are shown in the online Supplementary Information.
