# Supplementary material for: Impact of the COVID-19 pandemic on oncological care in Germany: rapid review
Source: J Cancer Res Clin Oncol. 2023 Jul 29;149(15):14329–40. doi: 10.1007/s00432-023-05063-9 (PMC10590309; doi:10.1007/s00432-023-05063-9)
Supplement: Supplementary file 1 — Supplementary file1 (ZIP 764 KB) [file 432_2023_5063_MOESM1_ESM.zip › Table_S10_results_registry_studies.docx]

#### Table S10. Oncological care during the COVID-19 pandemic: Cancer registry data (n = 8 studies)

| **Author, year** | **Data source and region** | **Pandemic period** | **Pre-pandemic period** | **Cancer type** | **Care aspects** | **Care during vs. pre-pandemic** | **Confounding factors** | **Prediction (modeling)** | **Risk of bias** |
| --- | --- | --- | --- | --- | --- | --- | --- | --- | --- |
| Erdmann 2021 ^1^; Erdmann 2022 ^2^; Schüz 2022 ^3^ | Nationwide German Childhood Cancer Registry | 2020 | 2015-2019 | any childhood | diagnosis | ↑ incidence overall | pandemic stage, cancer type,  socio-demographics, region |  | 0.75 (Erdmann 2021); 0.42 (Erdmann 2022) |
|  |  | 2020 | 2006-2019 |  |  |  |  | ↑ incidence leukemia 2-6 yrs | 0.06 (Schüz 2022) |
| Hölzel 2022 ^4^ | Nationwide German Breast Cancer Screening Program Registry | 2020 | 2018 | breast | other |  | cancer type, cancer stage | ↓ survival rate | Model 1: 0.25; Model 2: 0.19; Model 3: 0.25) |
| Justenhoven 2022 ^5^ | Rhineland Palatinate Cancer Registry | 2020-2021 | 2018-2019 | any | diagnosis, treatment, aftercare | ↓ incidence  ↓ surgery  ↔ radiotherapy  ↔ systemic  ↓ or ↔ aftercare | pandemic stage,  institution type |  | 0.50 |
| Piontek 2021 ^6^ | Saxony Cancer Registry | 2020 | 2017-2019 | any | diagnosis | ↓ or ↔ incidence | cancer type,  socio-demographics, region |  | 0.50 |
| Stang 2020 ^7^ | North Rhine Westphalia Cancer Registry | 2020 | 2019 | any | diagnosis | ↓ or ↔ detection | pandemic stage |  | 0.42 |
| Voigtländer 2021 ^8^ | Bavarian Cancer Registry | 2020 | 2019 | breast, prostate, colorectum, lung, skin | diagnosis, treatment | ↓ incidence stage I  ↔ incidence stage II-IV  ↓ or ↔ surgery  ↓ radiotherapy  ↔ systemic | pandemic stage, cancer type, cancer stage |  | 0.42 |

Note. Table includes modeling studies based on cancer registry data.

**References**

1. Erdmann F, Wellbrock M, Trubenbach C, Spix C, Schrappe M, Schuz J, et al. Impact of the COVID-19 pandemic on incidence, time of diagnosis and delivery of healthcare among paediatric oncology patients in Germany in 2020: Evidence from the German Childhood Cancer Registry and a qualitative survey. The Lancet Regional Health Europe. 2021 Oct;9:100188. doi: <https://dx.doi.org/10.1016/j.lanepe.2021.100188>.

2. Erdmann F, Spix C, Schrappe M, Borkhardt A, Schuz J. Temporal changes of the incidence of childhood cancer in Germany during the COVID-19 pandemic: Updated analyses from the German Childhood Cancer Registry. The Lancet Regional Health Europe. 2022 Jun;17:100398. doi: <https://dx.doi.org/10.1016/j.lanepe.2022.100398>.

3. Schuz J, Borkhardt A, Bouaoun L, Erdmann F. The impact of the COVID-19 pandemic on the future incidence of acute lymphoblastic leukaemia in children: Projections for Germany under a COVID-19 related scenario. International Journal of Cancer. 2022 07 01;151(1):153-5. doi: <https://dx.doi.org/10.1002/ijc.33992>.

4. Holzel D S-FGEJ. Estimation of the risk of progression of breast cancer after the COVID-19 lockdown. Deutsches Arzteblatt international. 2022;119(20):368-9. doi: 10.3238/arztebl.m2022.0165.

5. Justenhoven C, Rieger B. The impact of the Corona pandemic on reported data relating to cancer diagnoses, therapy, and follow-up: Analyses from the Rhineland–Palatinate Cancer Registry. Dtsch Arztebl Int. 2022;119:724-1. doi: 10.3238/arztebl.m2022.0299.

6. Piontek D, Klagges S, Schubotz B, Werner C, Wulff J. Documented New Cases of Cancer in the Clinical Cancer Registries of the German State of Saxony During the COVID-19 Pandemic. Deutsches Arzteblatt International. 2021 05 07;118(18):328-9. doi: <https://dx.doi.org/10.3238/arztebl.m2021.0216>.

7. Stang A, Kuhling L, Khil L, Kajuter H, Schutzendubel A, Mattauch V. Drop in Cancer Reporting by Pathologists in North Rhine-Westphalia, Germany, During the COVID-19 Lockdown. Deutsches Arzteblatt International. 2020 12 21;117(51-52):886-7. doi: <https://dx.doi.org/10.3238/arztebl.2020.0886>.

8. Voigtlander S, Hakimhashemi A, Inwald EC, Ortmann O, Gerken M, Klug SJ, et al. The Impact of the COVID-19 Pandemic on Cancer Incidence and Treatment by Cancer Stage in Bavaria, Germany. Deutsches Arzteblatt International. 2021 10 01;118(39):660-1. doi: <https://dx.doi.org/10.3238/arztebl.m2021.0329>.
