# Supplementary material for: Impact of the COVID-19 pandemic on oncological care in Germany: rapid review
Source: J Cancer Res Clin Oncol. 2023 Jul 29;149(15):14329–40. doi: 10.1007/s00432-023-05063-9 (PMC10590309; doi:10.1007/s00432-023-05063-9)
Supplement: Supplementary file 1 — Supplementary file1 (ZIP 764 KB) [file 432_2023_5063_MOESM1_ESM.zip › Table_S11_results_survey_studies.docx]

#### Table S11. Oncological care during the COVID-19 pandemic: Survey data (n = 21 studies)

| **Author, year** | **Data source** | **Region** | **Sample size** | **Pandemic period** | **Cancer type** | **Care aspects** | **Care perceptions for patients** | **Care perceptions for healthcare professionals** | **Confounding factors** | **Risk of bias score** |
| --- | --- | --- | --- | --- | --- | --- | --- | --- | --- | --- |
| Arndt 2022 ^1^ | healthcare professionals | nationwide | NR | 2020-2022 | any | any  treatment  aftercare | ↓ any care (restrictions) ↓ surgery ↓ psychosocial ↓ aftercare |  | pandemic stage, care type | 0.13 |
| Beller 2022 ^2^ | healthcare professionals | Lower Saxony | 200 | 2022 | any | any | ↓ any care (restrictions in diagnosis, psychosocial) ↔ any care (treatment, other- advanced and palliative care) | ↓ clinical management (decision conflicts, communication with patients, process quality) ↓ resources | care type | 0.50 |
| Brunner 2020 ^3^ | healthcare professionals | nationwide | 112 | 2020 | colorectal | treatment | ↓ surgery | ↓ clinical management (surgeons against delays in surgery) | region, institution type | 0.38 |
| Büntzel 2020 ^4^, Büntzel 2021 ^5^ | patients, healthcare professionals | nationwide | 752 | 2020 | any (most head, neck, breast) | any | ↓ any care (restrictions, fear of therapy changes) ↑ mental burden | ↓ clinical management (more effort to convince patients to undergo treatment) ↓ resources ↑ mental burden |  | 0.13 |
| Donath 2021 ^6^ | patients, healthcare professionals | Hesse | 65 | 2020 | any childhood | any | ↓ any care (consultations -40% pediatrician survey vs. -30% hospital administration data) | ↑ workload | cancer stage | 0.50 |
| Eckford 2021 ^7^ | patients | nationwide | 621 | 2020-2021 | any | any, treatment, aftercare | ↓ any care (fear of therapy changes) ↔ treatment ↓ psychosocial, aftercare ↑ mental burden |  | pandemic stage,  cancer stage, socio-demographics | 0.75 |
| Erdmann 2021 ^8^ | patients, healthcare professionals | nationwide | NR | 2020 | any childhood | any, diagnosis, treatment | ↓ any care (appointments, access for accompanying individuals) ↔ diagnosis, treatment, ↓ psychosocial | ↑ workload |  | 0.75 |
| Haier 2022 ^9^, Haier 2022 ^10^, Haier 2022 ^11^ | patients, healthcare professionals | nationwide | 1231 | 2020-2021 | any | any | ↓ any care (low satisfaction with information) ↔ treatment (decision uncertainty, treatment changes, delays) ↔ mental burden | ↔ clinical management (clinical processes, resources, delays) ↑ workload |  | 0.63 |
| Hajek 2021 ^12^ | patients | nationwide | 974 | 2020 | any | diagnosis | ↓ screening (postponed) |  | pandemic stage,  socio-demographics | 1 |
| Harke 2020 ^13^, Harke 2022 ^14^ | healthcare professionals | nationwide | NR | 2020 | uro-oncologic | treatment | ↓ treatment (postponed when less urgent) ↓ surgery (low-risk stage) ↔ surgeries, radiotherapy advanced stage |  | pandemic stage,  cancer type, cancer stage | 0.63 |
| Matuschek 2020 ^15^ | healthcare professionals | nationwide | NR | 2020 | any | any | ↓ any care (patient volume) |  |  | 0.38 |
| Micek 2022 ^16^, Teuscher 2022 ^17^ | patients | Berlin | 366 | 2020-2021 | skin | any, treatment | ↓ or ↔ any care (postponed appointments at patient request) ↔ treatment |  | pandemic stage,  cancer stage, treatment stage | 1 |
| Stöß 2020 ^18^ | healthcare professionals | nationwide | 148 | 2020 | any | any, treatment | ↓ any care (less consultations) ↓ surgeries outpatient clinics |  | institution type | 0.38 |
| Vu 2022 ^19^ | healthcare professionals | nationwide | 54 | 2020 | gynecologic | any, treatment | ↓ any care (access to practices, patient volume, postponed appointments) ↔ treatment (prioritization of therapies, treatment, systemic therapies) | ↓ clinical management (estimated financial losses) ↑ workload, ↑ mental burden |  | 0.13 |
| Walter 2021 ^20^ | patients (sample 1) | Bavaria | 67 | 2020 | thoracic | any | ↓ any care (access to care) ↔ any care (fear of changes to cancer care) |  |  | 0 |
| Walter 2022 ^21^ | patients, (sample 2) | Bavaria | 93 | 2020 | thoracic | any, treatment | ↓ any care (availability of ambulatory care), ↔ treatment |  |  | 0.50 |

**References**

1. Arndt V, Doege D, Frohling S, Albers P, Algul H, Bargou R, et al. Cancer care in German centers of excellence during the first 2 years of the COVID-19 pandemic. Journal of Cancer Research & Clinical Oncology. 2022 Oct 14;14:14. doi: <https://dx.doi.org/10.1007/s00432-022-04407-1>.

2. Beller J, Schafers J, Geyer S, Haier J, Epping J. Patterns of Changes in Oncological Care due to COVID-19: Results of a Survey of Oncological Nurses and Physicians from the Region of Hanover, Germany. Healthcare. 2022 Dec 22;10(1):22. doi: <https://dx.doi.org/10.3390/healthcare10010015>.

3. Brunner M, Krautz C, Kersting S, Weber GF, Stinner B, Benz SR, et al. Oncological colorectal surgery during the COVID-19pandemic-a national survey. International Journal of Colorectal Disease. 2020 Dec;35(12):2219-25. doi: <https://dx.doi.org/10.1007/s00384-020-03697-6>.

4. Buntzel J, Klein M, Keinki C, Walter S, Buntzel J, Hubner J. Oncology services in corona times: a flash interview among German cancer patients and their physicians. Journal of Cancer Research & Clinical Oncology. 2020 Oct;146(10):2713-5. doi: <https://dx.doi.org/10.1007/s00432-020-03249-z>.

5. Buntzel J, Micke O, Klein M, Buntzel J, Walter S, Keinki C, et al. Take care or "German Angst"? Lessons from cancer care during COVID-19 pandemic in spring 2020. Journal of Cancer Research & Clinical Oncology. 2021 Jul;147(7):2093-105. doi: <https://dx.doi.org/10.1007/s00432-020-03492-4>.

6. Donath H, Zielen S, Wittekindt B, Klingebiel T, Graf J, Eckrich M, et al. Effects of the SARS-CoV2-Lockdown on Pediatric Care in the Rhine-Main Area. Klinische Padiatrie. 2021 Jan;233(1):31-6. doi: <https://dx.doi.org/10.1055/a-1263-1467>.

7. Eckford RD, Gaisser A, Arndt V, Baumann M, Kludt E, Mehlis K, et al. The COVID-19 Pandemic and Cancer Patients in Germany: Impact on Treatment, Follow-Up Care and Psychological Burden. Frontiers in Public Health. 2021;9:788598. doi: <https://dx.doi.org/10.3389/fpubh.2021.788598>.

8. Erdmann F, Wellbrock M, Trubenbach C, Spix C, Schrappe M, Schuz J, et al. Impact of the COVID-19 pandemic on incidence, time of diagnosis and delivery of healthcare among paediatric oncology patients in Germany in 2020: Evidence from the German Childhood Cancer Registry and a qualitative survey. The Lancet Regional Health Europe. 2021 Oct;9:100188. doi: <https://dx.doi.org/10.1016/j.lanepe.2021.100188>.

9. Haier J, Beller J, Adorjan K, Bleich S, De Greck M, Griesinger F, et al. Decision Conflicts in Clinical Care during COVID-19: A Patient Perspective. Healthcare. 2022 May 31;10(6):31. doi: <https://dx.doi.org/10.3390/healthcare10061019>.

10. Haier J, Beller J, Adorjan K, Bleich S, de Greck M, Griesinger F, et al. Decision Conflicts in Clinical Care during COVID-19: A Multi-Perspective Inquiry. Healthcare. 2022 Sep 29;10(10):29. doi: <https://dx.doi.org/10.3390/healthcare10101914>.

11. Haier J, Beller J, Adorjan K, Bleich S, de Greck M, Griesinger F, et al. Differences in Stakeholders’ Perception of the Impact of COVID-19 on Clinical Care and Decision-Making. Cancers. 2022;14(17). doi: 10.3390/cancers14174317.

12. Hajek A, De Bock F, Huebl L, Kretzler B, König HH. Determinants of postponed cancer screening during the covid-19 pandemic: Evidence from the nationally representative covid-19 snapshot monitoring in Germany (cosmo). Risk Management and Healthcare Policy. 2021;14:3003-11. doi: 10.2147/RMHP.S297326.

13. Harke NN, Radtke JP, Hadaschik BA, Bach C, Berger FP, Blana A, et al. To defer or not to defer? A German longitudinal multicentric assessment of clinical practice in urology during the COVID-19 pandemic. PLoS ONE. 2020;15(9):e0239027. doi: <https://dx.doi.org/10.1371/journal.pone.0239027>.

14. Harke NN, Wagner C, Hermann RM, Hadaschik BA, Radtke JP, Altay-Langguth A, et al. Lessons learned after one year of COVID-19 from a urologist and radiotherapist view: A German survey on prostate cancer diagnosis and treatment. PLoS ONE. 2022;17(6):e0269827. doi: <https://dx.doi.org/10.1371/journal.pone.0269827>.

15. Matuschek C, Fischer JC, Combs SE, Fietkau R, Corradini S, Zanker K, et al. Measures of infection prevention and incidence of SARS-CoV-2 infections in cancer patients undergoing radiotherapy in Germany, Austria and Switzerland. Strahlentherapie und Onkologie. 2020 12;196(12):1068-79. doi: <https://dx.doi.org/10.1007/s00066-020-01681-1>.

16. Micek A, Diehl K, Teuscher M, Schaarschmidt ML, Sasama B, Ohletz J, et al. Melanoma care during one year pandemic in Berlin: decreasing appointment cancellations despite increasing COVID-19 concern. Journal der Deutschen Dermatologischen Gesellschaft. 2022 07;20(7):962-78. doi: <https://dx.doi.org/10.1111/ddg.14799>.

17. Teuscher M, Diehl K, Schaarschmidt ML, Weilandt J, Sasama B, Ohletz J, et al. Effects of the COVID-19 pandemic on care of melanoma patients in Berlin, Germany: the Mela-COVID survey. European Journal of Dermatology. 2022 07 01;31(4):521-9. doi: <https://dx.doi.org/10.1684/ejd.2021.4098>.

18. Stos C, Steffani M, Kohlhaw K, Rudroff C, Staib L, Hartmann D, et al. The COVID-19 pandemic: impact on surgical departments of non-university hospitals. BMC Surgery. 2020 Dec 03;20(1):313. doi: <https://dx.doi.org/10.1186/s12893-020-00970-x>.

19. Vu E, Schröder C, Dülk J, Stelmes JJ, Vu J, Schilling J, et al. Nationwide Survey of German Outpatient Gynecologic Oncology Practices during the Coronavirus Disease 2019 Pandemic: Reactions to the First Wave and Future Perspectives. Breast Care. 2022;17(3):257-63. doi: 10.1159/000518858.

20. Walter J, Sellmer L, Kahnert K, Zauber R, Syunyaeva Z, Kauffmann-Guerrero D, et al. Daily Routine and Access to Care: Initial Patient Reported Experiences at a German Lung Cancer Center during the COVID-19 Pandemic. Respiration. 2021;100(1):90-2. doi: <https://dx.doi.org/10.1159/000513849>.

21. Walter J, Sellmer L, Kahnert K, Kiefl R, Syunyaeva Z, Kauffmann-Guerrero D, et al. Consequences of the COVID-19 pandemic on lung cancer care and patient health in a German lung cancer center: results from a cross-sectional questionnaire. Respiratory Research. 2022 Jan 29;23(1):18. doi: <https://dx.doi.org/10.1186/s12931-022-01931-z>.
