# Supplementary material for: Impact of the COVID-19 pandemic on oncological care in Germany: rapid review
Source: J Cancer Res Clin Oncol. 2023 Jul 29;149(15):14329–40. doi: 10.1007/s00432-023-05063-9 (PMC10590309; doi:10.1007/s00432-023-05063-9)
Supplement: Supplementary file 1 — Supplementary file1 (ZIP 764 KB) [file 432_2023_5063_MOESM1_ESM.zip › Table_S12_results_reports.docx]

**Table S12. Oncological care during the COVID-19 pandemic: results from reports without peer-review.**

| **Organization, publication year** | **Data source** | **Publication format** | **Area of care** | **Reported indicators** | **Period covered** | **Reference year(s)** | **Observations compared to pre-pandemic reference period** |
| --- | --- | --- | --- | --- | --- | --- | --- |
| AOK and WIdO, 2021 [^1^](#_ENREF_1) | health claims data, AOK | book section | hospital; treatment | number of MDC 17 inpatient cases; number of tumor cases with resection | 01/2020-10/2020 | 2019 | *MDC 17 cases:* ↓  *cases with resection, CRC:*  01-02/2020: ↔  03-05/2020: ↓↓  06-09/2020: ↓  10/2020: ↓  *cases with resection, breast*:  01-02/2020: ↔  03-05/2020: ↓↓  06-09/2020: ↓  10/2020: ↔  *cases with resection, lung:*  01-02/2020: ↑  03-05/2020: ↓  06-09/2020: ↓  10/2020: ↓  *cases with resection, prostate:*  01-02/2020: ↑  03-05/2020: ↔  06-09/2020: ↓↓  10/2020: ↓↓ |
| bifg, 2021 [^2^](#_ENREF_2) | health claims data, BARMER | book section | hospital;  treatment | number of inpatient cases with tumor resection; monthly average admission rate per 10,000 insurants | 04/2020-06/2020 | 2017-2019 | colon: ↓↓  rectum: ↓↓  breast: ↓↓  lung: ↓↓  esophagus: ↓↓  stomach: ↓↓  pancreas: ↓  kidney: ↓↓  prostate: ↔  total rate: ↓↓ |
|  |  |  |  |  | 07/2020-10/2020 | 2017-2019 | colon: ↓↓  rectum: ↓↓  breast: ↔  lung: ↑  esophagus:  stomach: ↓↓  pancreas: ↑↑  kidney: ↑  prostate: ↓  total rate: ↓ |

| DKG, KKRBB, Ulm University Hospital and Zi, 2022 [^3^](#_ENREF_3) | health claims data,  AOK, InEK, Zi | book section | hospital;  medical practice;  treatment | number of hospital inpatient cancer cases, cancer cases in medical practice (total, CRC) | 01/2020-12/2020 | 2019 | *cancer total:*  hospital: ↓ AOK, ↓ InEK  medical practice: ↔ Zi  *CRC:*  hospital: ↓↓ AOK, ↓↓ InEK  medical practice: ↓ |
| --- | --- | --- | --- | --- | --- | --- | --- |
|  |  |  | hospital; treatment | number of inpatient cases with tumor resection (CRC) | 01/2020-12/2020 | 2019 | total 2020: ↓↓ AOK, ↓ InEK |
|  |  |  | hospital; diagnosis | number of inpatient colonoscopies; number of outpatient colonoscopies | 01/2020-12/2020 | 2019 | *inpatient:*  diagnostic: ↓↓  therapeutic: ↓↓  *outpatient:*  diagnostic: ↓↓  therapeutic: ↓↓ |
|  |  |  | medical practice; diagnosis | number of colonoscopies in medical practice | 01/2020-12/2020 | 2019 | screening: ↔ AOK, ↔ Zi  diagnostic: ↔ AOK, ↓ Zi  therapeutic: ↔ AOK, ↔ Zi |
|  | cancer registry data,  regional (BB, BE) | book section | diagnosis | stage at diagnosis (CRC) | 01/2020-12/2020 | 2019 | stage I: ↓  stage II: ↓  stage III: ↔  stage IV: ↔  unstaged: ↑↑ |
| WIdO, 2020 [^4^](#_ENREF_4) | health claims data, AOK | independent report | hospital;  medical practice;  diagnosis | number of cancer screening examinations per eligible population | 01/2020-12/2020 | 2007-2019 | cancer screening females: ↓  cancer screening males: ↓  colonoscopy (screening, diagnostic): ↓  mammography: ↓↓  skin: ↓↓ |
| WIdO, 2020 [^5^](#_ENREF_5) | health claims data, AOK | independent report | hospital; treatment | number of inpatient cases with tumor resection | 03/2020-04/2020 | 2019 | breast: ↑↑ primary, ↓↓↓ reconstructive, ↑ total  CRC: ↓↓↓ primary, ↓↓↓ secondary, ↓↓↓ total  brain: ↔  lung: ↓↓↓  pancreas: ↓  prostate: ↓ |
| WIdO 2021 [^6^](#_ENREF_6) | health claims data, AOK | press release | hospital;  treatment | number of inpatient cases with tumor resection | 03/2021-05/2021 | 2019 | breast: ↓  CRC: ↓↓ |
| WIdO, 2022 [^7^](#_ENREF_7) | health claims data, AOK | press release | hospital;  treatment | number of inpatient cases with tumor resection | 01/2022-05/2022 | 2019 | breast: ↓  CRC: ↓↓ |
| WINHO, 2021 [^8^](#_ENREF_8) | health claims data, SHI | article in membership magazine | medical practice; treatment | number of all patients; number of cancer patients with active illness; I.V. anti-cancer treatment; palliative treatment, etc. | Q2/2020 | Q3/2017-2019 | all patients: ↓  cancer patients: ↑  I.V. anti-cancer treatment: ↔  palliative treatment: ↔ |
| Zi, 2021 [^9^](#_ENREF_9) | health claims data, SHI | independent report | medical practice;  diagnosis;  treatment | number of hemato-oncology cases; number of “qualified oncology care” cases; number of cancer screenings; etc. | 01/2020-12/2020 | 2019 | indicators are reported for 14 individual 2020 time periods; annual totals are not reported (for 2020 and 2021 annual totals see: Zi, 2022 [^10^](#_ENREF_10)) |
| Zi, 2022 [^10^](#_ENREF_10) | health claims data, SHI | independent report | medical practice;  diagnosis;  treatment | number of hemato-oncology cases; number of “qualified oncology care”^‡^ cases; number of radiology cases for treatment of malignant neoplasms; number of cancer screenings | 01/2020-12/2021 | 2019 | “qualified oncology care”: ↔ 2020, ↔ 2021  hemato-oncology: ↔ 2020, ↑ 2021  radiology: ↔ 2020, ↓ 2021  *screening:*  colonoscopy: ↔ 2020, ↑ 2021  mammography: ↓ 2020, ↑ 2021  skin: ↓↓ 2020, ↓↓ 2021 |
| KKRBB, 2022 [^11^](#_ENREF_11) | cancer registry data,  regional (BE, BB) | news article on organizational website | diagnosis | preliminary data on reported lung cancers: stage at diagnosis; completeness of reports (%) | 01/2020-12/2020 | not reported | unquantified decline in early-stage, increase (esp. in Q3, Q4) in advanced-stage tumors;  completeness: > 95% |
| KKRBB, 2022 [^12^](#_ENREF_12) | cancer registry data,  regional (BE, BB) | guest commentary in membership magazine | diagnosis | number of reported female breast cancers; time between diagnosis and primary surgery (days); tumor size at pathological diagnosis (mm) | 01/2020-12/2020 | 2017-2019 | temporary decline in new cases; time between diagnosis and primary surgery unchanged; average tumor size at diagnosis +1 mm |
| KKR Sachsen, 2022 [^13^](#_ENREF_13) | cancer registry data, regional (SN) | independent report | diagnosis | absolute number of cases; number of cases per 100,000 population p.a.; stage at diagnosis, etc. | 01/2020-12/2020 | 2011-2019 | indicators are not explicitly compared against reference values, no statement on data quality or completeness (for COVID‑19-related effects on reported cancers in Saxony see: Piontek D, Klagges S, Schubotz B, Werner C, Wulff J (2021) Documented New Cases of Cancer in the Clinical Cancer Registries of the German State of Saxony During the COVID-19 Pandemic. Dtsch Arztebl Int 118:328-9. <https://doi.org/10.3238/arztebl.m2021.0216>) |
| DKFZ, DKG and DKH COVID-19 Task Force, 2020 [^14^](#_ENREF_14) | survey data | article in membership magazine | hospital;  diagnosis;  treatment;  aftercare;  other | number of hospitals that report discontinuation or restrictions of specific services; extent of reported restrictions | 03/2020-08/2020 | not reported | severe and lasting restrictions reported for: follow-up care, psycho-social care, nutrition counselling, therapeutic exercise, diagnostic imaging, systemic treatment of hematological disease, tumor surgery |
| DKFZ, DKG and DKH COVID-19 Task Force, 2021 [^15^](#_ENREF_15) | survey data,  unsolicited feedback | press release | hospital;  diagnosis;  treatment | experience of CCCs, non-university cancer centers and cancer information services | 03/2020-publication date | not reported | no survey results reported (for report of survey data see: DKFZ, DKG and DKH COVID-19 Task Force, 2020[^14^](#_ENREF_14)*)* |
| DKFZ, DKG and DKH COVID-19 Task Force, 2021 [^16^](#_ENREF_16) | survey data | press release | hospital;  treatment | experience of CCCs | 03/2020-publication date | not reported | 2 out of 3 hospitals have exhausted their (ICU) capacities to admit cancer patients |
| WIdO, 2021 [^17^](#_ENREF_17) | survey data | insert in subscription magazine | hospital;  medical practice;  diagnosis;  treatment | patient experience and utilization of cancer care since beginning of the pandemic | 03/2020-08/2021 | does not apply | queried cancer patients:  ° feel that the pandemic has negatively affected their anti-cancer treatment (11%);  ° have experienced pandemic-related cancelation or postponement of medical appointments by their healthcare provider (hospital: 9%; medical practice: 16%);  ° have cancelled or postponed medical appointments for fear of infection (hospital: 6%; medical practice: 16%);  eligible individuals:  ° have experienced cancelation or postponement of cancer screening appointments (mammography: 12%; colonoscopy: 5%; cervical: 9%; prostate: 10%; skin: 8%) |
| WINHO, 2021 [^18^](#_ENREF_18) | survey data | article in subscription magazine | medical practice;  treatment | experience of hematology-oncology practices | 04/2021-05/2021 | not reported | 38% of respondents report decline in patient numbers; 35% report increase in patient numbers; 31% report patient influx from nearby hospitals; 74% and 12% report provision of telemedicine/video telemedicine services; 4% report temporary closures due to quarantine regulations |

↓ indicator declined by ≥ 3% compared to reference period

↓↓ indicator declined by ≥ 10% compared to reference period

↓↓↓ indicator declined by ≥ 20% compared to reference period

↑ indicator increased by ≥ 3% compared to reference period

↑↑ indicator increased by ≥ 10% compared to reference period

↔ indicator stable compared to reference period (decline or increase by < 3%)

^‡^ refers to treatment according to the “Agreement on qualified ambulatory cancer care” (German: Vereinbarung über die qualifizierte ambulante Versorgung krebskranker Patienten, „Onkologie-Vereinbarung“); Central Federal Association of Health Insurance Funds and National Association of Statutory Health Insurance Physicians, 2009; <https://gkv-spitzenverband.de/krankenversicherung/aerztliche_versorgung/bundesmantelvertrag/anlagen_zum_bundesmantelvertrag/einzelne_anlagen_zum_bmv/bmv_anlage_7_onkologie.jsp>

abbreviations:

**AOK**: Local Health Insurance Funds (German: *Allgemeine Ortskrankenkasse*); **BB**: Brandenburg; **BE**: Berlin; **bifg**: BARMER Institute for health systems research (German: *BARMER Institut für Gesundheitssystemforschung*); **CCC**: Comprehensive Cancer Center; **DKFZ**: German Cancer Research Center (German: *Deutsches Krebsforschungszentrum*); **DKG**: German Cancer Society (German: *Deutsche Krebsgesellschaft*); **DKH**: German Cancer Aid (German: *Deutsche Krebshilfe*); **ICU**: intensive care unit; **KKR**: Clinical Cancer Registry or Clinical Cancer Registries (German: *Klinische(s) Krebsregister*); **KKRBB**: Berlin-Brandenburg Cancer Registry (German: *Klinisch-epidemiologisches Krebsregister Brandenburg-Berlin*); **MDC**: Major Diagnostic Category; **SHI**: statutory health insurance; SN: Saxony; **WIdO**: AOK Research Institute (German: *Wissenschaftliches Institut der AOK*); **WINHO**: Research Institute of Hematologists and Oncologists in Private Practice (German: *Wissenschaftliches Institut der niedergelassenen Hämatologen und Onkologen*); **Zi**: Central research institute of statutory health insurance physicians in Germany (German: *Zentralinstitut für die kassenärztliche Versorgung in Deutschland*)

**References**

1. Mostert C, Hentschker, C., Scheller-Kreinsen, D., Günster, C., , Malzahn J, Klauber J. Auswirkungen der Covid-19-Pandemie auf die Krankenhausleistungen im Jahr 2020. Krankenhaus-Report 2021 (report). <https://doi.org/10.1007/978-3-662-62708-2_16>. Accessed 26 October 2022.

2. Acar L, L’hoest H, Marschall U. Der Einfluss der Coronapandemie auf die medizinische Versorgung schwerwiegender Erkrankungen im Jahr 2020 (report). Gesundheitswesen aktuell, BARMER Institut für Gesundheitssystemforschung (bifg). <https://doi.org/10.30433/GWA2021-308>. Accessed 26 October 2022.

3. Rückher J, Mangiapane, S., Seufferlein, T., , Pflüger M, Wesselmann, S. Auswirkungen der Covid-19-Pandemie auf die onkologische Versorgung. Krankenhaus-Report 2022 (report). <https://doi.org/10.1007/978-3-662-64685-4_6>. Accessed 26 October 2022.

4. Tillmanns H, Schillinger G, Dräther H. WIdO-Report: Inanspruchnahme von Früherkennungsleistungen der gesetzlichen Krankenversicherung durch AOK-Versicherte im Erwachsenenalter (2009 bis 2020; report). Wissenschaftliches Institut der AOK (WIdO). <https://doi.org/10.4126/FRL01-006431137>. Accessed 26 October 2022.

5. Günster C, Drogan D, Hentschker C, Klauber J, Malzahn J, Schillinger G, et al. WIdO-Report: Entwicklung der Krankenhausfallzahlen während des Coronavirus-Lockdowns (report). Wissenschaftliches Institut der AOK (WIdO). <https://doi.org/10.4126/FRL01-006421684>. Accessed 26 October 2022.

6. Wissenschaftliches Institut der AOK (WIdO). WIdO-Analyse: Auch in der dritten Pandemiewelle wieder Fallzahlrückgänge in den Krankenhäusern (press release). <https://zmail.wido.de/news-presse/pressemitteilungen/2021/wido-analyse-auch-in-der-dritten-pandemiewelle-wieder-fallzahlrueckgaenge-in-den-krankenhaeusern/>. Accessed 26 October 2022.

7. Wissenschaftliches Institut der AOK (WIdO). Erneut starke Einbrüche bei Darmkrebs-Operationen in der Omikron-Welle (press release). <https://al.wido.de/news-presse/pressemitteilungen/2022/erneut-starke-einbrueche-bei-darmkrebs-operationen-in-der-omikron-welle/?L=0>. Accessed 26 October 2022.

8. Heidt V, Knauf W, Illmer T, Engel E, Goetzenich A (2021) Hämatoonkologische Praxen: Trotz Pandemie ambulant gut versorgt (magazine article). Dtsch Arztebl 118:A310-3. <www.aerzteblatt.de/lit0621>. Accessed 26 October 2022.

9. Mangiapane S, Zhu L, Kretschmann J, Czihal T, von Stillfried D. Veränderung der vertragsärztlichen Leistungsinanspruchnahme während der COVID-Krise - Tabellarischer Trendreport für das Jahr 2020 (report). Zentralinstitut für die kassenärztliche Versorgung in der Bundesrepublik Deutschland (Zi). <https://www.zi.de/fileadmin/Downloads/Service/Publikationen/Trendreport_4_Leistungsinanspruchnahme_COVID_2021-04-19.pdf>. Accessed 26 October 2022.

10. Mangiapane S, Zhu L, Kretschmann J, Czihal T, von Stillfried D. Veränderung der vertragsärztlichen Leistungsinanspruchnahme während der COVID-Krise – Tabellarischer Trendreport bis zum Ende des Jahres 2021 (report). Zentralinstitut für die kassenärztliche Versorgung in der Bundesrepublik Deutschland (Zi). <https://www.zi.de/fileadmin/Downloads/Service/Publikationen/Zi-TrendReport_2021-Q4_2022-06-10.pdf>. Accessed 26 October 2022.

11. Klinisches Krebsregister für Brandenburg und Berlin. Erste virtuelle Qualitätskonferenz des KKRBB zum Lungenkarzinom (report). <https://kkrbb.de/erste-virtuelle-qualitaetskonferenz-des-kkrbb-zum-lungenkarzinom/>. Accessed 26 October 2022.

12. Klinisches Krebsregister für Brandenburg und Berlin. Auswirkungen der COVID-19-Pandemie auf Diagnose und Therapie des Mammakarzinoms (magazine article). Brandenburgisches Ärzteblatt, Landesärztekammer Brandenburg. <https://www.laekb.de/documents/183A20B72FC.pdf>. Accessed 26 October 2022.

13. Klinische Krebsregister Sachsen. Jahresbericht der klinischen Krebsregister in Sachsen 2011 – 2020 (report). <https://www.krebsregister-sachsen.de/fileadmin/user_upload/dokumente/auswertungen/2022-07-06_Jahresbericht_KKR_Sachsen.pdf>. Accessed 26 October 2022.

14. Fröhling S, Arndt V (2020) Versorgung von Krebspatienten: Corona-Effekt in der Onkologie (magazine article). Dtsch Arztebl 117:A2234-42. <https://www.aerzteblatt.de/archiv/216717/Versorgung-von-Krebspatienten-Corona-Effekt-in-der-Onkologie>. Accessed 26 October 2022.

15. Deutsche Krebsgesellschaft Deutsche Krebshilfe Deutsches Krebsforschungszentrum. Versorgung von Krebspatient*innen hochgefährdet (press release). <https://www.krebsgesellschaft.de/deutsche-krebsgesellschaft-wtrl/willkommen/presse/pressemitteilungen-2021/versorgung-von-krebspatienten-hochgefaehrdet.html>. Accessed 26 October 2022.

16. Deutsche Krebsgesellschaft Deutsche Krebshilfe Deutsches Krebsforschungszentrum. Universitätskliniken fürchten Triage bei Krebspatient*innen (press release). <https://www.krebsgesellschaft.de/deutsche-krebsgesellschaft-wtrl/pressemitteilungen-2021/triage.html>. Accessed 26 October 2022.

17. Zok K. Gesundheitsverhalten und Erfahrungen mit der ambulantärztlichen Versorgung während der Covid-19-Pandemie - Ergebnisse einer bundesweiten Repräsentativbefragung (report). Wissenschaftliches Institut der AOK (WIdO). <https://www.wido.de/publikationen-produkte/widomonitor/widomonitor-2-2021/?L=0>. Accessed 26 October 2022.

18. Hermes-Moll K, Walawgo T, Richter M, Osburg S, Hempler I, Blattert L, et al. (2021) Ambulante Versorgung von Krebserkrankten: Umfrage unter hämatoonkologischen Schwerpunktpraxen zur COVID-19-Lage (magazine article). InFo Hämatologie + Onkologie 29:68-72. <https://www.springermedizin.de/covid-19/impfungen/umfrage-unter-haematoonkologischen-schwerpunktpraxen-zur-covid-1/19657926>. Accessed 26 October 2022.
