# Supplementary material for: Impact of the COVID-19 pandemic on oncological care in Germany: rapid review
Source: J Cancer Res Clin Oncol. 2023 Jul 29;149(15):14329–40. doi: 10.1007/s00432-023-05063-9 (PMC10590309; doi:10.1007/s00432-023-05063-9)
Supplement: Supplementary file 1 — Supplementary file1 (ZIP 764 KB) [file 432_2023_5063_MOESM1_ESM.zip › Table_S4_search_reports.docx]

**Table S4. Search strategy for reports without peer-review**

| **Organization** | **Homepage** | **Type** |
| --- | --- | --- |
| BARMER Institut für Gesundheits­systemforschung (bifg) | <https://www.bifg.de/> | *research institute, statutory health insurance funds* |
| Wissenschaftliches Institut der AOK (WIdO) | <https://www.wido.de/> | *research institute, statutory health insurance funds* |
| Wissenschaftliches Institut der Niedergelassenen Hämatologen und Onkologen (WINHO) | <https://winho.de/> | *research institute, association of hematologists and oncologists in private practice* |
| Wissenschaftliches Institut der PKV (WIP) | <https://www.wip-pkv.de/> | *research institute, private health insurers association* |
| Zentralinstitut für die kassenärztliche Versorgung in Deutschland (Zi) | <https://www.zi.de/> | *research institute, association of statutory health insurance physicians* |
| Deutsche Krebsgesellschaft (DKG) | https://www.krebsgesellschaft.de/ | *oncology/hematology specialist society* |
| Deutsche Gesellschaft für Hämatologie und  Medizinische Onkologie (DGHO) | <https://www.dgho.de/> | *oncology/hematology specialist society* |
| Bayerisches Krebsregister | <https://www.lgl.bayern.de/gesundheit/krebsregister/> | *cancer registry* |
| Bremer Krebsregister | <https://www.krebsregister.bremen.de/> | *cancer registry* |
| Hamburgisches Krebsregister | <https://www.hamburg.de/> | *cancer registry* |
| Hessisches Krebsregister | <https://hessisches-krebsregister.de/> | *cancer registry* |
| Klinische Krebsregister Sachsen | <https://www.krebsregister-sachsen.de/> | *cancer registry* |
| Klinische Krebsregister Sachsen-Anhalt | <https://www.kkr-lsa.de/> | *cancer registry* |
| Klinisches Krebsregister für Brandenburg und Berlin | <https://kkrbb.de/> | *cancer registry* |
| Klinisches Krebsregister Thüringen | <https://www.krebsregister-thueringen.de/> | *cancer registry* |
| Krebsregister Baden-Württemberg | <https://www.krebsregister-bw.de/> | *cancer registry* |
| Krebsregister Mecklenburg-Vorpommern | <https://www.kkr-mv.de/> | *cancer registry* |
| Krebsregister Niedersachsen | <https://www.krebsregister-niedersachsen.de/> | *cancer registry* |
| Krebsregister Rheinland-Pfalz | <https://www.krebsregister-rlp.de/> | *cancer registry* |
| Krebsregister Saarland | <https://krebsregister.saarland.de/> | *cancer registry* |
| Krebsregister Schleswig-Holstein | <https://www.krebsregister-sh.de/> | *cancer registry* |
| Landeskrebsregister Nordrhein-Westfalen | <https://www.landeskrebsregister.nrw/> | *cancer registry* |

Note. To identify relevant non-peer-reviewed literature, we searched the websites of selected organizations for publications that reported quantitative or qualitative data on cancer care in Germany collected between 1 March 2020 and 31 October 2022.

The search was conducted between 31 October 2022 and 15 November 2022 and updated on 13 December 2022.

We searched the websites of Germany-based research institutes of statutory and private health insurers (bifg, WIdO, WIP), research institutes of physician associations (WINHO, Zi), regional cancer registries, and hematology/oncology specialist societies (DGHO, DKG).

Relevant reports (n=17) were identified.

A manual search of cited references identified one additional non-peer-reviewed report that met the inclusion criteria.

In total, 18 relevant reports were identified and included in this review.
