# Supplementary material for: Impact of the COVID-19 pandemic on oncological care in Germany: rapid review
Source: J Cancer Res Clin Oncol. 2023 Jul 29;149(15):14329–40. doi: 10.1007/s00432-023-05063-9 (PMC10590309; doi:10.1007/s00432-023-05063-9)
Supplement: Supplementary file 1 — Supplementary file1 (ZIP 764 KB) [file 432_2023_5063_MOESM1_ESM.zip › Table_S5_RoB_instruments.docx]

**Supplementary Information**

## Table S5. Risk of bias assessment instruments.

| **Studies with peer-review** | **Instrument name** | **Source** | **Notes** |
| --- | --- | --- | --- |
| Studies with survey data | Checklist for Analytical Cross Sectional Studies | Moola S, Munn Z, Tufanaru C, Aromataris E, Sears K, Sfetcu R, Currie M, Lisy K, Qureshi R, Mattis P, Mu P. Chapter 7: Systematic reviews of etiology and risk. In: Aromataris E, Munn Z (Editors). JBI Manual for Evidence Synthesis. JBI, 2020. Available from  [https://synthesismanual.jbi.global](https://synthesismanual.jbi.global/).  <https://doi.org/10.46658/JBIMES-20-08> | Assessment performed as explained in the source |
| Modeling studies | Bespoke risk of bias instrument for modeling studies | - Burns, J., Movsisyan, A., Stratil, J. M., Biallas, R. L., Coenen, M., Emmert-Fees, K. M., Geffert, K., Hoffmann, S., Horstick, O., Laxy, M., Klinger, C., Kratzer, S., Litwin, T., Norris, S., Pfadenhauer, L. M., von Philipsborn, P., Sell, K., Stadelmaier, J., Verboom, B., Voss, S., … Rehfuess, E. (2021). International travel-related control measures to contain the COVID-19 pandemic: a rapid review. The Cochrane database of systematic reviews, 3(3), CD013717. <https://doi.org/10.1002/14651858.CD013717.pub2> - Jaime Caro, J., Eddy, D. M., Kan, H., Kaltz, C., Patel, B., Eldessouki, R., Briggs, A. H., & ISPOR-AMCP-NPC Modeling CER Task Forces (2014). Questionnaire to assess relevance and credibility of modeling studies for informing health care decision making: an ISPOR-AMCP-NPC Good Practice Task Force report. Value in health : the journal of the International Society for Pharmacoeconomics and Outcomes Research, 17(2), 174–182. <https://doi.org/10.1016/j.jval.2014.01.003> - Philips, Z., Bojke, L., Sculpher, M., Claxton, K., & Golder, S. (2006). Good practice guidelines for decision-analytic modelling in health technology assessment: a review and consolidation of quality assessment. PharmacoEconomics, 24(4), 355–371. <https://doi.org/10.2165/00019053-200624040-00006> | Description of the composition of the instrument: Questions from two sources (Philips et al., 2006 and Caro et al., 2014) were combined in this instrument to assess the risk of bias of the included modeling studies. This instrument consists of the domains model structure, data, uncertainty, external and internal validation and transparency. Except for a few additional questions in the model structure and data domains, the instrument is identical to that of Burns et al., 2021. |
| Studies with registry or administrative data | Bespoke risk of bias tool for studies with registry or administrative data | - <https://www.nhlbi.nih.gov/health-topics/study-quality-assessment-tools> - Sterne JAC, Hernán MA, Reeves BC, Savović J, Berkman ND, Viswanathan M, Henry D, Altman DG, Ansari MT, Boutron I, Carpenter JR, Chan AW, Churchill R, Deeks JJ, Hróbjartsson A, Kirkham J, Jüni P, Loke YK, Pigott TD, Ramsay CR, Regidor D, Rothstein HR, Sandhu L, Santaguida PL, Schünemann HJ, Shea B, Shrier I, Tugwell P, Turner L, Valentine JC, Waddington H, Waters E, Wells GA, Whiting PF, Higgins JPT. ROBINS-I: a tool for assessing risk of bias in non-randomized studies of interventions. BMJ 2016; 355; i4919. - Sterne JAC, Hernán MA, McAleenan A, Reeves BC, Higgins JPT. Chapter 25: Assessing risk of bias in a non-randomized study. In: Higgins JPT, Thomas J, Chandler J, Cumpston M, Li T, Page MJ, Welch VA (editors). Cochrane Handbook for Systematic Reviews of Interventions version 6.3 (updated February 2022). Cochrane, 2022. Available from [www.training.cochrane.org/handbook.](http://www.training.cochrane.org/handbook.) | Description of the composition of the instrument: This risk of bias assessment tool for registry and administrative data consists of questions from both the NHLBI and ROBINS-I instruments, covering bias domains outlined in section 25.5 of the Cochrane manual. |
