# Supplementary material for: Impact of the COVID-19 pandemic on oncological care in Germany: rapid review
Source: J Cancer Res Clin Oncol. 2023 Jul 29;149(15):14329–40. doi: 10.1007/s00432-023-05063-9 (PMC10590309; doi:10.1007/s00432-023-05063-9)
Supplement: Supplementary file 1 — Supplementary file1 (ZIP 764 KB) [file 432_2023_5063_MOESM1_ESM.zip › Table_S6_excluded_studies.docx]

**Supplementary Information**

## Table S6. List of excluded studies and sources of included studies.

| **Study status** | **Studies, n** | **Citation** |
| --- | --- | --- |
| **Included studies with peer-review** | **59** | ^1-59^ |
| from manual searches of bibliographies | 4/59 | ^4, 34, 40, 59^ |
| from searches of study registers | 1/59 | ^29^ |
| **Included reports without peer-review** | **18** | ^60-77^ |
| from manual search of bibliographies | 1/18 | ^68^ |
| **Excluded studies with peer-review after full-text screening** | **10** | ^78-87^ |
| Exclusion 2: no focus of impact of COVID-pandemic on cancer care | 6/10 | ^78-80, 82-84^ |
| Exclusion 3: no focus on Germany | 4/10 | ^81, 85-87^ |

1. Arndt V, Doege D, Frohling S, Albers P, Algul H, Bargou R, et al. (2022) Cancer care in German centers of excellence during the first 2 years of the COVID-19 pandemic. J Cancer Res Clin Oncol 14:14. <https://doi.org/10.1007/s00432-022-04407-1>

2. Balakirski G, Michalowitz AL, Kreuter A, Hofmann SC (2022) Long-term effects of the COVID-19 pandemic on malignant melanoma: increased lymph node metastases in two German dermatology clinics. J Eur Acad Dermatol Venereol 36:e762-e4. <https://doi.org/10.1111/jdv.18337>

3. Balk M, Rupp R, Craveiro AV, Allner M, Grundtner P, Eckstein M, et al. (2022) The COVID-19 pandemic and its consequences for the diagnosis and therapy of head and neck malignancies. Eur Rev Med Pharmacol Sci 26:284-90. <https://doi.org/10.26355/eurrev_202201_27779>

4. Bartella AK, Halama D, Kamal M, Hahnel S, Sander AK, Pausch NC, et al. (2021) Impact of COVID-19 on Oral and Maxillofacial Surgery: Preliminary Results After the Curfew. J Craniofac Surg 32:e305-e8. <https://doi.org/10.1097/scs.0000000000007062>

5. Beller J, Schafers J, Geyer S, Haier J, Epping J (2022) Patterns of Changes in Oncological Care due to COVID-19: Results of a Survey of Oncological Nurses and Physicians from the Region of Hanover, Germany. Healthcare 10:22. <https://doi.org/10.3390/healthcare10010015>

6. Bollmann A, Hohenstein S, Pellissier V, Stengler K, Reichardt P, Ritz JP, et al. (2021) Utilization of in- and outpatient hospital care in Germany during the Covid-19 pandemic insights from the German-wide Helios hospital network. PLoS ONE 16:e0249251. <https://doi.org/10.1371/journal.pone.0249251>

7. Brunner M, Krautz C, Kersting S, Weber GF, Stinner B, Benz SR, et al. (2020) Oncological colorectal surgery during the COVID-19 pandemic- a national survey. Int J Colorectal Dis 35:2219-25. <https://doi.org/10.1007/s00384-020-03697-6>

8. Buntzel J, Klein M, Keinki C, Walter S, Buntzel J, Hubner J (2020) Oncology services in corona times: a flash interview among German cancer patients and their physicians. J Cancer Res Clin Oncol 146:2713-5. <https://doi.org/10.1007/s00432-020-03249-z>

9. Buntzel J, Micke O, Klein M, Buntzel J, Walter S, Keinki C, et al. (2021) Take care or "German Angst"? Lessons from cancer care during COVID-19 pandemic in spring 2020. J Cancer Res Clin Oncol 147:2093-105. <https://doi.org/10.1007/s00432-020-03492-4>

10. Dienemann T, Brennfleck F, Dejaco A, Grutzmann R, Binder J, Krautz C, et al. (2021) Collateral effects of the SARS-CoV-2 pandemic on oncologic surgery in Bavaria. BMC Surgery 21:411. <https://doi.org/10.1186/s12893-021-01404-y>

11. Diers J, Acar L, Baum P, Flemming S, Kastner C, Germer CT, et al. (2021) Fewer Operations for Cancer in Germany During the First Wave of COVID-19 in 2020-A Cohort Study and Time-Series Analysis. Dtsch Arztebl Int 118:481-2. <https://doi.org/10.3238/arztebl.m2021.0265>

12. Diers J, Acar L, Wagner JC, Baum P, Hankir M, Flemming S, et al. (2022) Cancer diagnosis is one quarter lower than the expected cancer incidence in the first year of COVID-19 pandemic in Germany: A retrospective register-based cohort study. Cancer Commun 42:673-6. <https://doi.org/10.1002/cac2.12314>

13. Donath H, Zielen S, Wittekindt B, Klingebiel T, Graf J, Eckrich M, et al. (2021) Effects of the SARS-CoV2-Lockdown on Pediatric Care in the Rhine-Main Area. Klin Padiatr 233:31-6. <https://doi.org/10.1055/a-1263-1467>

14. Eckford RD, Gaisser A, Arndt V, Baumann M, Kludt E, Mehlis K, et al. (2021) The COVID-19 Pandemic and Cancer Patients in Germany: Impact on Treatment, Follow-Up Care and Psychological Burden. Front Public Health 9:788598. <https://doi.org/10.3389/fpubh.2021.788598>

15. Erdmann F, Spix C, Schrappe M, Borkhardt A, Schuz J (2022) Temporal changes of the incidence of childhood cancer in Germany during the COVID-19 pandemic: Updated analyses from the German Childhood Cancer Registry. Lancet Reg 17:100398. <https://doi.org/10.1016/j.lanepe.2022.100398>

16. Erdmann F, Wellbrock M, Trubenbach C, Spix C, Schrappe M, Schuz J, et al. (2021) Impact of the COVID-19 pandemic on incidence, time of diagnosis and delivery of healthcare among paediatric oncology patients in Germany in 2020: Evidence from the German Childhood Cancer Registry and a qualitative survey. Lancet Reg 9:100188. <https://doi.org/10.1016/j.lanepe.2021.100188>

17. Fauser D, Banaschak H, Zollmann P, Streibelt M, Bethge M (2022) Impact of the SARS-CoV-2 Pandemic on the Utilization of Cancer Rehabilitation: a Difference-in-Differences Analysis. Rehabilitation 24:24. <https://doi.org/10.1055/a-1936-4083>

18. Gremke N, Griewing S, Felgentreff M, Kostev K, Kalder M (2022) Impact of the Coronavirus Disease 2019 (COVID-19) Pandemic on Cervical Cancer Screening in Gynecological Practices in Germany. Cancers 14. <https://doi.org/10.3390/cancers14194820>

19. Griewing S, Wagner U, Lingenfelder M, Fischer R, Kalder M (2022) Chronological development of in-patient oncology in times of COVID-19: a retrospective analysis of hospitalized oncology and COVID-19 patients of a German University Hospital. J Cancer Res Clin Oncol 30:30. <https://doi.org/10.1007/s00432-022-04044-8>

20. Griewing S, Wagner U, Lingenfelder M, Heinis S, Schieffer B, Markus B, et al. (2022) Impact of the COVID-19 Pandemic on Delivery of Gynecology and Obstetrics Services at a Maximum Care University Hospital in Germany. Geburtshilfe Frauenheilkd 82:427-40. <https://doi.org/10.1055/a-1687-9674>

21. Gschnell M, Federspiel P, Wolf R (2021) COVID-19-Lockdown Impacts Medical Care - A Retrospective Analysis of the First Wave at a University Outpatient Clinic in Spring 2020. Aktuelle Derm 47:552-7. <https://doi.org/10.1055/a-1660-4813>

22. Haier J, Beller J, Adorjan K, Bleich S, De Greck M, Griesinger F, et al. (2022) Decision Conflicts in Clinical Care during COVID-19: A Patient Perspective. Healthcare 10:31. <https://doi.org/10.3390/healthcare10061019>

23. Haier J, Beller J, Adorjan K, Bleich S, de Greck M, Griesinger F, et al. (2022) Decision Conflicts in Clinical Care during COVID-19: A Multi-Perspective Inquiry. Healthcare 10:29. <https://doi.org/10.3390/healthcare10101914>

24. Haier J, Beller J, Adorjan K, Bleich S, de Greck M, Griesinger F, et al. (2022) Differences in Stakeholders’ Perception of the Impact of COVID-19 on Clinical Care and Decision-Making. Cancers 14. <https://doi.org/10.3390/cancers14174317>

25. Hajek A, De Bock F, Huebl L, Kretzler B, König HH (2021) Determinants of postponed cancer screening during the covid-19 pandemic: Evidence from the nationally representative covid-19 snapshot monitoring in Germany (cosmo). Risk Manag Healthc Policy 14:3003-11. <https://doi.org/10.2147/RMHP.S297326>

26. Harke NN, Radtke JP, Hadaschik BA, Bach C, Berger FP, Blana A, et al. (2020) To defer or not to defer? A German longitudinal multicentric assessment of clinical practice in urology during the COVID-19 pandemic. PLoS ONE 15:e0239027. <https://doi.org/10.1371/journal.pone.0239027>

27. Harke NN, Wagner C, Hermann RM, Hadaschik BA, Radtke JP, Altay-Langguth A, et al. (2022) Lessons learned after one year of COVID-19 from a urologist and radiotherapist view: A German survey on prostate cancer diagnosis and treatment. PLoS ONE 17:e0269827. <https://doi.org/10.1371/journal.pone.0269827>

28. Heimes D, Müller LK, Schellin A, Naujokat H, Graetz C, Schwendicke F, et al. (2021) Consequences of the COVID-19 pandemic and governmental containment policies on the detection and therapy of oral malignant lesions—a retrospective, multicenter cohort study from Germany. Cancers 13. <https://doi.org/10.3390/cancers13122892>

29. Holzel D, Schubert-Fritschle G, Engel J (2022) Estimation of the risk of progression of breast cancer after the COVID-19 lockdown. Dtsch Arztebl Int 119:368-9. <https://doi.org/10.3238/arztebl.m2022.0165>

30. Hunger R, König V, Stillger R, Mantke R (2022) Impact of the COVID-19 pandemic on delays in surgical procedures in Germany: a multi-center analysis of an administrative registry of 176,783 patients. Patient Saf Surg 16. <https://doi.org/10.1186/s13037-022-00331-y>

31. Jacob L, Kalder M, Kostev K (2022) Decrease in the number of patients diagnosed with cancer during the COVID-19 pandemic in Germany. J Cancer Res Clin Oncol 148:3117-23. <https://doi.org/10.1007/s00432-022-03922-5>

32. Jacob L, Loosen SH, Kalder M, Luedde T, Roderburg C, Kostev K (2021) Impact of the COVID-19 pandemic on cancer diagnoses in general and specialized practices in Germany. Cancers 13:1-11. <https://doi.org/10.3390/cancers13030408>

33. Jördens MS, Loosen SH, Seraphin T, Luedde T, Kostev K, Roderburg C (2021) Impact of the COVID-19 Pandemic on Consultations and Diagnoses in Gastroenterology Practices in Germany. Front Med 8. <https://doi.org/10.3389/fmed.2021.684032>

34. Justenhoven C, Rieger B (2022) The impact of the Corona pandemic on reported data relating to cancer diagnoses, therapy, and follow-up: Analyses from the Rhineland–Palatinate Cancer Registry. Dtsch Arztebl Int 119:724-1. <https://doi.org/10.3238/arztebl.m2022.0299>

35. Kaltofen T, Hagemann F, Harbeck N, Wuerstlein R, Kost BP, Burges A, et al. (2022) Changes in gynecologic and breast cancer diagnoses during the first wave of the COVID-19 pandemic: analysis from a tertiary academic gyneco-oncological center in Germany. Arch Gynecol Obstet 305:713-8. <https://doi.org/10.1007/s00404-021-06211-7>

36. Kapsner LA, Kampf MO, Seuchter SA, Gruendner J, Gulden C, Mate S, et al. (2020) Reduced Rate of Inpatient Hospital Admissions in 18 German University Hospitals During the COVID-19 Lockdown. Front Public Health 8:594117. <https://doi.org/10.3389/fpubh.2020.594117>

37. Kirchberg J, Rentsch A, Klimova A, Vovk V, Hempel S, Folprecht G, et al. (2021) Influence of the First Wave of the COVID-19 Pandemic on Cancer Care in a German Comprehensive Cancer Center. Front Public Health 9:750479. <https://doi.org/10.3389/fpubh.2021.750479>

38. Kleemann J, Meissner M, Ozistanbullu D, Balaban U, Old O, Kippenberger S, et al. (2022) Impact of the Covid-19 pandemic on melanoma and non-melanoma skin cancer inpatient treatment in Germany - a nationwide analysis. J Eur Acad Dermatol Venereol 36:1766-73. <https://doi.org/10.1111/jdv.18217>

39. Kourtidis S, Munst J, Hofmann VM (2022) Effects of the COVID-19 Pandemic on Head and Neck Cancer Stage and Treatment Duration. Cureus 14:e26744. <https://doi.org/10.7759/cureus.26744>

40. Kuhlen R, Schmithausen D, Winklmair C, Schick J, Scriba P (2020) The Effects of the COVID-19 Pandemic and Lockdown on Routine Hospital Care for Other Illnesses. Dtsch Arztebl Int 117:488-9. <https://doi.org/10.3238/arztebl.2020.0488>

41. Matuschek C, Fischer JC, Combs SE, Fietkau R, Corradini S, Zanker K, et al. (2020) Measures of infection prevention and incidence of SARS-CoV-2 infections in cancer patients undergoing radiotherapy in Germany, Austria and Switzerland. Strahlenther Onkol 196:1068-79. <https://doi.org/10.1007/s00066-020-01681-1>

42. Medenwald D, Brunner T, Christiansen H, Kisser U, Mansoorian S, Vordermark D, et al. (2022) Shift of radiotherapy use during the first wave of the COVID-19 pandemic? An analysis of German inpatient data. Strahlenther Onkol 198:334-45. <https://doi.org/10.1007/s00066-021-01883-1>

43. Micek A, Diehl K, Teuscher M, Schaarschmidt ML, Sasama B, Ohletz J, et al. (2022) Melanoma care during one year pandemic in Berlin: decreasing appointment cancellations despite increasing COVID-19 concern. J Dtsch Dermatol Ges 20:962-78. <https://doi.org/10.1111/ddg.14799>

44. Michalowsky B, Hoffmann W, Bohlken J, Kostev K (2021) Effect of the COVID-19 lockdown on disease recognition and utilisation of healthcare services in the older population in Germany: a cross-sectional study. Age Ageing 50:317-25. <https://doi.org/10.1093/ageing/afaa260>

45. Piontek D, Klagges S, Schubotz B, Werner C, Wulff J (2021) Documented New Cases of Cancer in the Clinical Cancer Registries of the German State of Saxony During the COVID-19 Pandemic. Dtsch Arztebl Int 118:328-9. <https://doi.org/10.3238/arztebl.m2021.0216>

46. Reichardt P, Bollmann A, Hohenstein S, Glass B, Untch M, Reichardt A, et al. (2021) Decreased Incidence of Oncology Admissions in 75 Helios Hospitals in Germany during the COVID-19 Pandemic. Oncol Res Treat 44:71-5. <https://doi.org/10.1159/000512935>

47. Riemann S, Speck I, Gerstacker K, Becker C, Knopf A (2021) Collateral damage of the COVID-19 pandemic: an alarming decline in critical procedures in otorhinolaryngology in a German university hospital. Eur Arch Oto-Rhino-L 278:3417-23. <https://doi.org/10.1007/s00405-020-06519-1>

48. Rupa R, Sass B, Morales Lema MA, Nimsky C, Voellger B (2020) The Demand for Elective Neurosurgery at a German University Hospital during the First Wave of COVID-19. Healthcare 8:13. <https://doi.org/10.3390/healthcare8040483>

49. Schuz J, Borkhardt A, Bouaoun L, Erdmann F (2022) The impact of the COVID-19 pandemic on the future incidence of acute lymphoblastic leukaemia in children: Projections for Germany under a COVID-19 related scenario. Int J Cancer 151:153-5. <https://doi.org/10.1002/ijc.33992>

50. Stang A, Kuhling L, Khil L, Kajuter H, Schutzendubel A, Mattauch V (2020) Drop in Cancer Reporting by Pathologists in North Rhine-Westphalia, Germany, During the COVID-19 Lockdown. Dtsch Arztebl Int 117:886-7. <https://doi.org/10.3238/arztebl.2020.0886>

51. Stos C, Steffani M, Kohlhaw K, Rudroff C, Staib L, Hartmann D, et al. (2020) The COVID-19 pandemic: impact on surgical departments of non-university hospitals. BMC Surgery 20:313. <https://doi.org/10.1186/s12893-020-00970-x>

52. Struck JP, Schnoor M, Schulze A, Hupe MC, Ozimek T, Oppolzer IA, et al. (2022) Impact of COVID-19 crisis on medical care of patients with metastasized uro-oncologic disease under systemic cancer therapy: a multicenter study in German university hospitals. World J Urol 40:409-18. <https://doi.org/10.1007/s00345-021-03868-2>

53. Teuscher M, Diehl K, Schaarschmidt ML, Weilandt J, Sasama B, Ohletz J, et al. (2022) Effects of the COVID-19 pandemic on care of melanoma patients in Berlin, Germany: the Mela-COVID survey. Eur J Dermatol 31:521-9. <https://doi.org/10.1684/ejd.2021.4098>

54. Voigtlander S, Hakimhashemi A, Inwald EC, Ortmann O, Gerken M, Klug SJ, et al. (2021) The Impact of the COVID-19 Pandemic on Cancer Incidence and Treatment by Cancer Stage in Bavaria, Germany. Dtsch Arztebl Int 118:660-1. <https://doi.org/10.3238/arztebl.m2021.0329>

55. Vu E, Schröder C, Dülk J, Stelmes JJ, Vu J, Schilling J, et al. (2022) Nationwide Survey of German Outpatient Gynecologic Oncology Practices during the Coronavirus Disease 2019 Pandemic: Reactions to the First Wave and Future Perspectives. Breast Care 17:257-63. <https://doi.org/10.1159/000518858>

56. Walter J, Sellmer L, Kahnert K, Kiefl R, Syunyaeva Z, Kauffmann-Guerrero D, et al. (2022) Consequences of the COVID-19 pandemic on lung cancer care and patient health in a German lung cancer center: results from a cross-sectional questionnaire. Respir Res 23:18. <https://doi.org/10.1186/s12931-022-01931-z>

57. Walter J, Sellmer L, Kahnert K, Zauber R, Syunyaeva Z, Kauffmann-Guerrero D, et al. (2021) Daily Routine and Access to Care: Initial Patient Reported Experiences at a German Lung Cancer Center during the COVID-19 Pandemic. Respiration 100:90-2. <https://doi.org/10.1159/000513849>

58. Wang R, Helf C, Tizek L, Neuhauser R, Eyerich K, Zink A, et al. (2020) The Impact and Consequences of SARS-CoV-2 Pandemic on a Single University Dermatology Outpatient Clinic in Germany. Int J Environ Res Public Health 17:26. <https://doi.org/10.3390/ijerph17176182>

59. Ziegler E, Hill J, Lieske B, Klein J, dem OVK, Kofahl C (2022) Empowerment in cancer patients: Does peer support make a difference? A systematic review. Psychooncology 31:683-704. <https://doi.org/10.1002/pon.5869>

60. Wissenschaftliches Institut der AOK (WIdO). WIdO-Analyse: Auch in der dritten Pandemiewelle wieder Fallzahlrückgänge in den Krankenhäusern (press release). <https://zmail.wido.de/news-presse/pressemitteilungen/2021/wido-analyse-auch-in-der-dritten-pandemiewelle-wieder-fallzahlrueckgaenge-in-den-krankenhaeusern/>. Accessed 26 October 2022. 2021 July 29, 2021. Report No.

61. Deutsche Krebsgesellschaft Deutsche Krebshilfe Deutsches Krebsforschungszentrum. Universitätskliniken fürchten Triage bei Krebspatient*innen (press release). <https://www.krebsgesellschaft.de/deutsche-krebsgesellschaft-wtrl/pressemitteilungen-2021/triage.html>. Accessed 26 October 2022. 2021 December 21, 2021. Report No.

62. Deutsche Krebsgesellschaft Deutsche Krebshilfe Deutsches Krebsforschungszentrum. Versorgung von Krebspatient*innen hochgefährdet (press release). <https://www.krebsgesellschaft.de/deutsche-krebsgesellschaft-wtrl/willkommen/presse/pressemitteilungen-2021/versorgung-von-krebspatienten-hochgefaehrdet.html>. Accessed 26 October 2022. 2021 April 19, 2021. Report No.

63. Wissenschaftliches Institut der AOK (WIdO). Erneut starke Einbrüche bei Darmkrebs-Operationen in der Omikron-Welle (press release). <https://al.wido.de/news-presse/pressemitteilungen/2022/erneut-starke-einbrueche-bei-darmkrebs-operationen-in-der-omikron-welle/?L=0>. Accessed 26 October 2022. 2022 September 8, 2022. Report No.

64. Klinisches Krebsregister für Brandenburg und Berlin. Erste virtuelle Qualitätskonferenz des KKRBB zum Lungenkarzinom (report). <https://kkrbb.de/erste-virtuelle-qualitaetskonferenz-des-kkrbb-zum-lungenkarzinom/>. Accessed 26 October 2022. 2022 April 1, 2022. Report No.

65. Klinische Krebsregister Sachsen. Jahresbericht der klinischen Krebsregister in Sachsen 2011 – 2020 (report). <https://www.krebsregister-sachsen.de/fileadmin/user_upload/dokumente/auswertungen/2022-07-06_Jahresbericht_KKR_Sachsen.pdf>. Accessed 26 October 2022. 2022 July 2022. Report No.

66. Klinisches Krebsregister für Brandenburg und Berlin. Auswirkungen der COVID-19-Pandemie auf Diagnose und Therapie des Mammakarzinoms (magazine article). Brandenburgisches Ärzteblatt, Landesärztekammer Brandenburg. <https://www.laekb.de/documents/183A20B72FC.pdf>. Accessed 26 October 2022. 2022 October 1, 2022. Report No.

67. Acar L, L’hoest H, Marschall U. Der Einfluss der Coronapandemie auf die medizinische Versorgung schwerwiegender Erkrankungen im Jahr 2020 (report). Gesundheitswesen aktuell, BARMER Institut für Gesundheitssystemforschung (bifg). <https://doi.org/10.30433/GWA2021-308>. Accessed 26 October 2022. 2021. doi: 10.30433/GWA2021-308.

68. Fröhling S, Arndt V (2020) Versorgung von Krebspatienten: Corona-Effekt in der Onkologie (magazine article). Dtsch Arztebl 117:A2234-42. <https://www.aerzteblatt.de/archiv/216717/Versorgung-von-Krebspatienten-Corona-Effekt-in-der-Onkologie>. Accessed 26 October 2022.

69. Günster C, Drogan D, Hentschker C, Klauber J, Malzahn J, Schillinger G, et al. WIdO-Report: Entwicklung der Krankenhausfallzahlen während des Coronavirus-Lockdowns (report). Wissenschaftliches Institut der AOK (WIdO). <https://doi.org/10.4126/FRL01-006421684>. Accessed 26 October 2022. Berlin: 2020 June 26, 2020. . Report No.

70. Heidt V, Knauf W, Illmer T, Engel E, Goetzenich A (2021) Hämatoonkologische Praxen: Trotz Pandemie ambulant gut versorgt (magazine article). Dtsch Arztebl 118:A310-3. <www.aerzteblatt.de/lit0621>. Accessed 26 October 2022.

71. Hermes-Moll K, Walawgo T, Richter M, Osburg S, Hempler I, Blattert L, et al. (2021) Ambulante Versorgung von Krebserkrankten: Umfrage unter hämatoonkologischen Schwerpunktpraxen zur COVID-19-Lage (magazine article). InFo Hämatologie + Onkologie 29:68-72. <https://www.springermedizin.de/covid-19/impfungen/umfrage-unter-haematoonkologischen-schwerpunktpraxen-zur-covid-1/19657926>. Accessed 26 October 2022.

72. Mangiapane S, Zhu L, Kretschmann J, Czihal T, von Stillfried D. Veränderung der vertragsärztlichen Leistungsinanspruchnahme während der COVID-Krise - Tabellarischer Trendreport für das Jahr 2020 (report). Zentralinstitut für die kassenärztliche Versorgung in der Bundesrepublik Deutschland (Zi). <https://www.zi.de/fileadmin/Downloads/Service/Publikationen/Trendreport_4_Leistungsinanspruchnahme_COVID_2021-04-19.pdf>. Accessed 26 October 2022. 2021 April 16, 2021. . Report No.

73. Mangiapane S, Zhu L, Kretschmann J, Czihal T, von Stillfried D. Veränderung der vertragsärztlichen Leistungsinanspruchnahme während der COVID-Krise – Tabellarischer Trendreport bis zum Ende des Jahres 2021 (report). Zentralinstitut für die kassenärztliche Versorgung in der Bundesrepublik Deutschland (Zi). <https://www.zi.de/fileadmin/Downloads/Service/Publikationen/Zi-TrendReport_2021-Q4_2022-06-10.pdf>. Accessed 26 October 2022. 2022 June 8, 2022. . Report No.

74. Mostert C, Hentschker, C., Scheller-Kreinsen, D., Günster, C., , Malzahn J, Klauber J. Auswirkungen der Covid-19-Pandemie auf die Krankenhausleistungen im Jahr 2020. Krankenhaus-Report 2021 (report). <https://doi.org/10.1007/978-3-662-62708-2_16>. Accessed 26 October 2022. 2021. doi: 10.1007/978-3-662-62708-2_16.

75. Rückher J, Mangiapane, S., Seufferlein, T., , Pflüger M, Wesselmann, S. Auswirkungen der Covid-19-Pandemie auf die onkologische Versorgung. Krankenhaus-Report 2022 (report). <https://doi.org/10.1007/978-3-662-64685-4_6>. Accessed 26 October 2022. 2022. doi: 10.1007/978-3-662-64685-4_6.

76. Tillmanns H, Schillinger G, Dräther H. WIdO-Report: Inanspruchnahme von Früherkennungsleistungen der gesetzlichen Krankenversicherung durch AOK-Versicherte im Erwachsenenalter (2009 bis 2020; report). Wissenschaftliches Institut der AOK (WIdO). <https://doi.org/10.4126/FRL01-006431137>. Accessed 26 October 2022. Berlin: Wissenschaftliches Institut der AOK (WIdO). Report. , 2022 January, 2022. . Report No. doi: 10.4126/FRL01-006431137.

77. Zok K. Gesundheitsverhalten und Erfahrungen mit der ambulantärztlichen Versorgung während der Covid-19-Pandemie - Ergebnisse einer bundesweiten Repräsentativbefragung (report). Wissenschaftliches Institut der AOK (WIdO). <https://www.wido.de/publikationen-produkte/widomonitor/widomonitor-2-2021/?L=0>. Accessed 26 October 2022. 2021.

78. Akuamoa-Boateng D, Wegen S, Ferdinandus J, Marksteder R, Baues C, Marnitz S (2020) Managing patient flows in radiation oncology during the COVID-19 pandemic: Reworking existing treatment designs to prevent infections at a German hot spot area University Hospital. Strahlenther Onkol 196:1080-5. <https://doi.org/10.1007/s00066-020-01698-6>

79. Babic B, Datta RR, Schroder W, Schiffmann LM, Schmidt T, Bruns CJ, et al. (2021) Impact of COVID-19 on oncological surgery of the upper gastrointestinal tract. Chirurg 92:929-35. <https://doi.org/10.1007/s00104-021-01489-4>

80. Bauerle A, Musche V, Schmidt K, Schweda A, Fink M, Weismuller B, et al. (2021) Mental Health Burden of German Cancer Patients before and after the Outbreak of COVID-19: Predictors of Mental Health Impairment. Int J Environ Res Public Health 18:26. <https://doi.org/10.3390/ijerph18052318>

81. Freudenberg LS, Dittmer U, Herrmann K (2020) Impact of COVID-19 on Nuclear Medicine in Germany, Austria and Switzerland: An International Survey in April 2020. Nuclear-Medizin 59:294-9. <https://doi.org/10.1055/a-1163-3096>

82. Freudenberg LS, Essler M, Herrmann K (2021) Impact of COVID-19 on Nuclear Medicine Procedures in Germany 2020 - Results of a National Survey. Nuclear-Medizin 60:210-5. <https://doi.org/10.1055/a-1446-7641>

83. Klingenstein A, Samel C, Hintschich C (2022) Potential Delay of Diagnosing Infraorbital Skin Tumors Due to Coverage by Face Masks During the COVID-19 Pandemic: An Observational Study. Clin Ophthalmol 16:3581-7. <https://doi.org/10.2147/OPTH.S384217>

84. Kriegmair MC, Speck T, Schneider AW, Volkmer B, Michel MS (2021) Urological care in practices and clinics during the corona virus pandemic in Germany. Urologe 60:318-30. <https://doi.org/10.1007/s00120-021-01458-z>

85. Krug S, Garbe J, König S, Ungewiss H, Michl P, Rinke A, et al. (2020) Professional assessment of the impact of covid-19 on handling net patients. J Clin Med 9:1-10. <https://doi.org/10.3390/jcm9113633>

86. Pergolini I, Demir IE, Stoss C, Emmanuel K, Rosenberg R, Friess H, et al. (2021) Effects of COVID-19 Pandemic on the Treatment of Pancreatic Cancer: A Perspective from Central Europe. Dig Surg 38:158-65. <https://doi.org/10.1159/000513157>

87. Reuter-Oppermann M, Muller-Polyzou R, Wirtz H, Georgiadis A (2020) Influence of the pandemic dissemination of COVID-19 on radiotherapy practice: A flash survey in Germany, Austria and Switzerland. PLoS ONE 15:e0233330. <https://doi.org/10.1371/journal.pone.0233330>
