# Supplementary material for: Impact of the COVID-19 pandemic on oncological care in Germany: rapid review
Source: J Cancer Res Clin Oncol. 2023 Jul 29;149(15):14329–40. doi: 10.1007/s00432-023-05063-9 (PMC10590309; doi:10.1007/s00432-023-05063-9)
Supplement: Supplementary file 1 — Supplementary file1 (ZIP 764 KB) [file 432_2023_5063_MOESM1_ESM.zip › Table_S9_results_administrative_studies.docx]

#### Table S9. Oncological care during the COVID-19 pandemic: Administrative data (n = 32 studies)

| **Author, year** | **Data source and region** | **Pandemic period** | **Pre-pandemic** | **Cancer type** | **Care provision** | **Care aspects** | **Care during vs. pre-pandemic** | **Confounding factors** | **Risk of bias** |
| --- | --- | --- | --- | --- | --- | --- | --- | --- | --- |
| **Hospital, clinical practice, disease database data** | | | | | | | | | |
| Balakirski 2022 ^1^ | Helios group | 2020-2021 | 2019 | skin | inpatient | diagnosis, treatment | ↔ or ↓ detection (more metastases) ↔ surgery | pandemic stage | 0.75 |
| Bollmann 2021 ^2^ | Helios group | 2020 | 2019 | any | any | any, other | ↓ any (length of stay, admissions: ↓ inpatient, ↑ outpatient)  ↓ other (↑ in-hospital mortality without COVID) | pandemic stage, care provision | 0.58 |
| Reichardt 2020 ^3^ | Helios group | 2020 | 2019 | any | any | any | ↓ any (all admissions) ↔ any (admissions by cancer type) | pandemic stage, cancer type / stage, sociodemographics, region | 0.50 |
| Balk 2022 ^4^ | Bavaria | 2020-2021 | 2019-2020 | head, neck | inpatient | detection,  treatment | ↔ any (detection, treatment start, cancer stage) ↓ any (detection or diagnosis of metastases) | cancer type / stage, sociodemographics, region | 0.50 |
| Dienemann 2021 ^5^ | Bavaria | 2020 | 2017-2019 | gastrointestinal, peritoneal, endocrine | inpatient | treatment | ↓ surgery | pandemic stage, institution type | 0.75 |
| Kaltofen 2022 ^6^ | Bavaria | 2020 | 2019 | gynecologic, breast | any | diagnosis | ↓ diagnosis (new diagnoses, higher tumor stages, more cases with tumor-associated symptoms, lower age at first diagnosis) |  | 0.58 |
| Wang 2020 ^7^ | Bavaria | 2020 | 2015-2019 | skin | outpatient | any, diagnosis | ↓ any (less consultations) ↓ diagnosis |  | 0.75 |
| Ziegler 2022 ^8^ | Bavaria | 2020-2021 | 2019 | skin | outpatient | diagnosis | ↓ or ↔ diagnosis (less demand for screening, higher or same tumor penetration depth) | cancer type / stage | 0.50 |
| Bartella 2021 ^9^ | Saxony | 2020 | 2019 | head, neck, skin | inpatient | treatment | ↓ or ↔ surgery (low risk cancelled, financial loss of approximately 100,256.50 Euro/week) ↓ or ↔ postsurgical care (more postsurgical isolation due to lockdown and visitor restrictions) | pandemic stage, cancer type / stage | 0.25 |
| Kirchberg 2021 ^10^ | Saxony | 2020 | 2014-2019 | gastrointestinal | any | diagnosis | ↓ or ↔ tumor board presentations (more curative and palliative, primary tumors, local recurrent tumors, metastases, less curable cases) | pandemic stage, cancer type / stage | 0.58 |
| Donath 2021 ^11^ | Hesse | 2020 | 2019 | any childhood | any | any, diagnosis | ↓ any care (less consultations, hospital admissions, longer length of stay) ↔ or ↓ diagnosis (less first diagnoses) | diagnosis stage (first or any) | 0.50 |
| Griewing 2022 ^12^, Griewing 2022 ^13^ | Hesse | 2020 | 2016-2019 | breast, ovary | any | diagnosis | ↓ diagnosis (most types, some types increased) | pandemic stage, cancer type / stage | 0.58 |
|  |  | 2020-2021 | 2017-2019 | any | inpatient | any | ↓ hospitalizations (most decline in cancer types detectable by screening) | cancer type | 0.67 |
| Gschnell 2021 ^14^ | Hesse | 2020 | 2018-2019 | skin | outpatient | any | ↓ any (less appointments and first introductions during lockdown, lower patient age) | pandemic stage | 0.67 |
| Rupa 2020 ^15^ | Hesse | 2020 | 2019 | any | outpatient | treatment | ↔ surgery (elective neurosurgery) |  | 0.50 |
| Heimes 2021 ^16^ | nationwide | 2020 | 2018-2019 | oral | any | diagnosis, treatment | ↔ diagnosis (stage IV: less during restrictions, more after restrictions lifted) ↔ treatment (time to treatment shorter during restrictions and same after restrictions lifted) | pandemic stage, cancer stage region | 0.42 |
| Hunger 2022 ^17^ | nationwide | 2020-2021 | 2019-2020 | colorectal | inpatient | treatment | ↓ surgery | cancer type | 0.42 |
| Kleemann 2022 ^18^ | nationwide | 2020-2021 | 2019 | skin | inpatient | any, treatment | ↓ any (less cases) ↓ surgery | cancer stage | 0.67 |
| Kourtidis 2022 ^19^ | Berlin | 2020-2021 | 2019 | head, neck | any | diagnosis | ↔ diagnosis (stages and intervals to treatment) | cancer stage | 0.67 |
| Kuhlen 2020 ^20^ | nationwide | 2020 | 2019 | lung, colorectal, stomach, breast, bladder, prostata | inpatient | any | ↓ any (less cases, shorter length of stay) | cancer type | 0.25 |
| Riemann 2021 ^21^ | Baden Württemberg | 2020 | 2020 | head, neck | any | diagnosis, treatment | ↓ diagnosis ↔ surgery |  | 0.58 |
| Struck 2022 ^22^ | nationwide | 2020 | none | uro‑oncologic | any | treatment, aftercare | ↓ treatment, follow-up (changed, delayed or cancelled) | cancer type, sociodemographics | 0.58 |
| Jacob 2021 ^23^, Jacob 2022 ^24^ | Disease Analyzer database | 2020 | 2019 | any | outpatient | diagnosis | ↓ diagnosis | pandemic stage, cancer type, institution type, sociodemographics | 0.67 |
|  |  | 2020-2021 |  |  |  |  |  |  |  |
| Jördens 2021 ^25^ | Disease Analyzer database | 2020 | 2019 | gastrointestinal | outpatient | any | ↓ any (consultations, cases: less during restrictions, more after restrictions lifted) | pandemic stage, sociodemographics | 0.83 |
| Michalowsky 2020 ^26^ | Disease Analyzer database | 2020 | 2019 | any | outpatient | diagnosis | ↓ diagnosis |  | 0.67 |
| Kapsner 2020 ^27^ | MIRACUM group | 2020 | 2018-2019 | lung, brain | inpatient | treatment | ↓ surgery | cancer type | 0.50 |
| Medenwald 2022 ^28^ | MIRACUM group | 2020 | 2018-2019 | head, neck, cervical | inpatient | any, treatment | ↓ or ↔ any ↓ or ↔ surgery ↓ or ↔ radiotherapy | pandemic stage, cancer type | 0.58 |
| **Insurance data** | | | | | | | | | |
| Diers 2021 ^29^, Diers 2022 ^30^ | BARMER Insurance Company | 2020 | 2017-2019 | any | any | treatment | ↓ surgery (increase after restrictions lifted) | pandemic stage,  cancer type / stage, sociodemographics | 0.58 |
|  |  | 2020-2021 |  |  |  | diagnosis, treatment | ↓ incidence (any, cancers detected by screening) ↓ treatment (invasive, surgery, chemo- and radiotherapy for any stage and metastases) |  |  |
| Fauser 2022 ^31^ | Statutory Pension | 2020 | 2019 | female genital, colon, prostate, urological | rehabilitation | aftercare | ↓ rehabilitation |  | 0.67 |
| Gremke 2022 ^32^ | Statutory Health | 2020-2021 | 2018-2019 | cervical cancer | outpatient | diagnosis | ↓ screening (gynecological clinical and cytological examination) | sociodemographics | 0.75 |

**References**

1. Balakirski G, Michalowitz AL, Kreuter A, Hofmann SC. Long-term effects of the COVID-19 pandemic on malignant melanoma: increased lymph node metastases in two German dermatology clinics. Journal of the European Academy of Dermatology & Venereology. 2022 10;36(10):e762-e4. doi: <https://dx.doi.org/10.1111/jdv.18337>.

2. Bollmann A, Hohenstein S, Pellissier V, Stengler K, Reichardt P, Ritz JP, et al. Utilization of in- and outpatient hospital care in Germany during the Covid-19 pandemic insights from the German-wide Helios hospital network. PLoS ONE. 2021;16(3):e0249251. doi: <https://dx.doi.org/10.1371/journal.pone.0249251>.

3. Reichardt P, Bollmann A, Hohenstein S, Glass B, Untch M, Reichardt A, et al. Decreased Incidence of Oncology Admissions in 75 Helios Hospitals in Germany during the COVID-19 Pandemic. Oncology Research and Treatment. 2021;44(3):71-5. doi: <https://dx.doi.org/10.1159/000512935>.

4. Balk M, Rupp R, Craveiro AV, Allner M, Grundtner P, Eckstein M, et al. The COVID-19 pandemic and its consequences for the diagnosis and therapy of head and neck malignancies. European Review for Medical & Pharmacological Sciences. 2022 01;26(1):284-90. doi: <https://dx.doi.org/10.26355/eurrev_202201_27779>.

5. Dienemann T, Brennfleck F, Dejaco A, Grutzmann R, Binder J, Krautz C, et al. Collateral effects of the SARS-CoV-2 pandemic on oncologic surgery in Bavaria. BMC Surgery. 2021 Dec 04;21(1):411. doi: <https://dx.doi.org/10.1186/s12893-021-01404-y>.

6. Kaltofen T, Hagemann F, Harbeck N, Wuerstlein R, Kost BP, Burges A, et al. Changes in gynecologic and breast cancer diagnoses during the first wave of the COVID-19 pandemic: analysis from a tertiary academic gyneco-oncological center in Germany. Archives of Gynecology & Obstetrics. 2022 03;305(3):713-8. doi: <https://dx.doi.org/10.1007/s00404-021-06211-7>.

7. Wang R, Helf C, Tizek L, Neuhauser R, Eyerich K, Zink A, et al. The Impact and Consequences of SARS-CoV-2 Pandemic on a Single University Dermatology Outpatient Clinic in Germany. International Journal of Environmental Research & Public Health [Electronic Resource]. 2020 08 26;17(17):26. doi: <https://dx.doi.org/10.3390/ijerph17176182>.

8. Ziegler P. [Impact of the COVID-19 pandemic on skin cancer screening : Results of a large dermatology practice in Bavaria's largest county by area]. Hautarzt. 2022 Mar;73(3):212-5. doi: 10.1007/s00105-022-04941-6.

9. Bartella AK, Halama D, Kamal M, Hahnel S, Sander AK, Pausch NC, et al. Impact of COVID-19 on Oral and Maxillofacial Surgery: Preliminary Results After the Curfew. J Craniofac Surg. 2021 May 1;32(3):e305-e8. doi: 10.1097/scs.0000000000007062.

10. Kirchberg J, Rentsch A, Klimova A, Vovk V, Hempel S, Folprecht G, et al. Influence of the First Wave of the COVID-19 Pandemic on Cancer Care in a German Comprehensive Cancer Center. Frontiers in Public Health. 2021;9:750479. doi: <https://dx.doi.org/10.3389/fpubh.2021.750479>.

11. Donath H, Zielen S, Wittekindt B, Klingebiel T, Graf J, Eckrich M, et al. Effects of the SARS-CoV2-Lockdown on Pediatric Care in the Rhine-Main Area. Klinische Padiatrie. 2021 Jan;233(1):31-6. doi: <https://dx.doi.org/10.1055/a-1263-1467>.

12. Griewing S, Wagner U, Lingenfelder M, Heinis S, Schieffer B, Markus B, et al. Impact of the COVID-19 Pandemic on Delivery of Gynecology and Obstetrics Services at a Maximum Care University Hospital in Germany. Geburtshilfe und Frauenheilkunde. 2022;82(4):427-40. doi: 10.1055/a-1687-9674.

13. Griewing S, Wagner U, Lingenfelder M, Fischer R, Kalder M. Chronological development of in-patient oncology in times of COVID-19: a retrospective analysis of hospitalized oncology and COVID-19 patients of a German University Hospital. Journal of Cancer Research & Clinical Oncology. 2022 Jun 30;30:30. doi: <https://dx.doi.org/10.1007/s00432-022-04044-8>.

14. Gschnell M, Federspiel P, Wolf R. COVID-19-Lockdown Impacts Medical Care - A Retrospective Analysis of the First Wave at a University Outpatient Clinic in Spring 2020. Aktuelle Dermatologie. 2021;47(12):552-7. doi: 10.1055/a-1660-4813.

15. Rupa R, Sass B, Morales Lema MA, Nimsky C, Voellger B. The Demand for Elective Neurosurgery at a German University Hospital during the First Wave of COVID-19. Healthcare. 2020 Nov 13;8(4):13. doi: <https://dx.doi.org/10.3390/healthcare8040483>.

16. Heimes D, Müller LK, Schellin A, Naujokat H, Graetz C, Schwendicke F, et al. Consequences of the COVID-19 pandemic and governmental containment policies on the detection and therapy of oral malignant lesions—a retrospective, multicenter cohort study from germany. Cancers. 2021;13(12). doi: 10.3390/cancers13122892.

17. Hunger R, König V, Stillger R, Mantke R. Impact of the COVID-19 pandemic on delays in surgical procedures in Germany: a multi-center analysis of an administrative registry of 176,783 patients. Patient Safety in Surgery. 2022;16(1). doi: 10.1186/s13037-022-00331-y.

18. Kleemann J, Meissner M, Ozistanbullu D, Balaban U, Old O, Kippenberger S, et al. Impact of the Covid-19 pandemic on melanoma and non-melanoma skin cancer inpatient treatment in Germany - a nationwide analysis. Journal of the European Academy of Dermatology & Venereology. 2022 Oct;36(10):1766-73. doi: <https://dx.doi.org/10.1111/jdv.18217>.

19. Kourtidis S, Munst J, Hofmann VM. Effects of the COVID-19 Pandemic on Head and Neck Cancer Stage and Treatment Duration. Cureus. 2022 Jul;14(7):e26744. doi: <https://dx.doi.org/10.7759/cureus.26744>.

20. Kuhlen R, Schmithausen D, Winklmair C, Schick J, Scriba P. The Effects of the COVID-19 Pandemic and Lockdown on Routine Hospital Care for Other Illnesses. Dtsch Arztebl Int. 2020 Jul 6;117(27-28):488-9. doi: 10.3238/arztebl.2020.0488.

21. Riemann S, Speck I, Gerstacker K, Becker C, Knopf A. Collateral damage of the COVID-19 pandemic: an alarming decline in critical procedures in otorhinolaryngology in a German university hospital. European Archives of Oto-Rhino-Laryngology. 2021 Sep;278(9):3417-23. doi: <https://dx.doi.org/10.1007/s00405-020-06519-1>.

22. Struck JP, Schnoor M, Schulze A, Hupe MC, Ozimek T, Oppolzer IA, et al. Impact of COVID-19 crisis on medical care of patients with metastasized uro-oncologic disease under systemic cancer therapy: a multicenter study in German university hospitals. World Journal of Urology. 2022 Feb;40(2):409-18. doi: <https://dx.doi.org/10.1007/s00345-021-03868-2>.

23. Jacob L, Loosen SH, Kalder M, Luedde T, Roderburg C, Kostev K. Impact of the COVID-19 pandemic on cancer diagnoses in general and specialized practices in Germany. Cancers. 2021;13(3):1-11. doi: 10.3390/cancers13030408.

24. Jacob L, Kalder M, Kostev K. Decrease in the number of patients diagnosed with cancer during the COVID-19 pandemic in Germany. Journal of Cancer Research & Clinical Oncology. 2022 Nov;148(11):3117-23. doi: <https://dx.doi.org/10.1007/s00432-022-03922-5>.

25. Jördens MS, Loosen SH, Seraphin T, Luedde T, Kostev K, Roderburg C. Impact of the COVID-19 Pandemic on Consultations and Diagnoses in Gastroenterology Practices in Germany. Frontiers in Medicine. 2021;8. doi: 10.3389/fmed.2021.684032.

26. Michalowsky B, Hoffmann W, Bohlken J, Kostev K. Effect of the COVID-19 lockdown on disease recognition and utilisation of healthcare services in the older population in Germany: a cross-sectional study. Age & Ageing. 2021 02 26;50(2):317-25. doi: <https://dx.doi.org/10.1093/ageing/afaa260>.

27. Kapsner LA, Kampf MO, Seuchter SA, Gruendner J, Gulden C, Mate S, et al. Reduced Rate of Inpatient Hospital Admissions in 18 German University Hospitals During the COVID-19 Lockdown. Frontiers in Public Health. 2020;8:594117. doi: <https://dx.doi.org/10.3389/fpubh.2020.594117>.

28. Medenwald D, Brunner T, Christiansen H, Kisser U, Mansoorian S, Vordermark D, et al. Shift of radiotherapy use during the first wave of the COVID-19 pandemic? An analysis of German inpatient data. Strahlentherapie und Onkologie. 2022 04;198(4):334-45. doi: <https://dx.doi.org/10.1007/s00066-021-01883-1>.

29. Diers J, Acar L, Baum P, Flemming S, Kastner C, Germer CT, et al. Fewer Operations for Cancer in Germany During the First Wave of COVID-19 in 2020-A Cohort Study and Time-Series Analysis. Deutsches Arzteblatt International. 2021 07 12;118(27-28):481-2. doi: <https://dx.doi.org/10.3238/arztebl.m2021.0265>.

30. Diers J, Acar L, Wagner JC, Baum P, Hankir M, Flemming S, et al. Cancer diagnosis is one quarter lower than the expected cancer incidence in the first year of COVID-19 pandemic in Germany: A retrospective register-based cohort study. Cancer Communications. 2022 07;42(7):673-6. doi: <https://dx.doi.org/10.1002/cac2.12314>.

31. Fauser D, Banaschak H, Zollmann P, Streibelt M, Bethge M. [Impact of the SARS-CoV-2 Pandemic on the Utilization of Cancer Rehabilitation: a Difference-in-Differences Analysis]. Rehabilitation. 2022 Oct 24;24:24. doi: <https://dx.doi.org/10.1055/a-1936-4083>.

32. Gremke N, Griewing S, Felgentreff M, Kostev K, Kalder M. Impact of the Coronavirus Disease 2019 (COVID-19) Pandemic on Cervical Cancer Screening in Gynecological Practices in Germany. Cancers. 2022;14(19). doi: 10.3390/cancers14194820.
